# Dynamics and stoichiometry of a regulated enhancer-binding protein in live *Escherichia coli* cells

**DOI:** 10.1038/ncomms2997

**Published:** 2013-06-14

**Authors:** Parul Mehta, Goran Jovanovic, Tchern Lenn, Andreas Bruckbauer, Christoph Engl, Liming Ying, Martin Buck

**Affiliations:** 1 Department of Life Sciences, Imperial College London, London SW7 2AZ, UK; 2 Lymphocyte Interaction Laboratory, London Research Institute, Cancer Research UK, London WC2A 3LY, UK; 3 Faculty of Medicine, Molecular Medicine, National Heart and Lung Institute, Imperial College London, London SW7 2AZ, UK; 4 Present address: Lawrence Berkeley National laboratory, University of California, Berkeley, USA; 5 Present address: Skirball Institute of Biomolecular Medicine, New York University Medical School, New York, USA

## Abstract

Bacterial enhancer-dependent transcription systems support major adaptive responses and offer a singular paradigm in gene control analogous to complex eukaryotic systems. Here we report new mechanistic insights into the control of one-membrane stress-responsive bacterial enhancer-dependent system. Using millisecond single-molecule fluorescence microscopy of live cells we determine the localizations, two-dimensional diffusion dynamics and stoichiometries of complexes of the bacterial enhancer-binding ATPase PspF during its action at promoters as regulated by inner membrane interacting negative controller PspA. We establish that a stable repressive PspF–PspA complex is located in the nucleoid, transiently communicating with the inner membrane via PspA. The PspF as a hexamer stably binds only one of the two *psp* promoters at a time, suggesting that *psp* promoters will fire asynchronously and cooperative interactions of PspF with the basal transcription complex influence dynamics of the PspF hexamer–DNA complex and regulation of the *psp* promoters.

## Abstract (web summary)

Cellular adaptive responses require temporal and spatial control of key regulatory protein complexes. Mehta *et al.* describe the dynamic interaction of a transcriptional activator mediating membrane stress response in *E. coli* with its negative regulator, the cell membrane and the transcription machinery.

## Graphical abstract

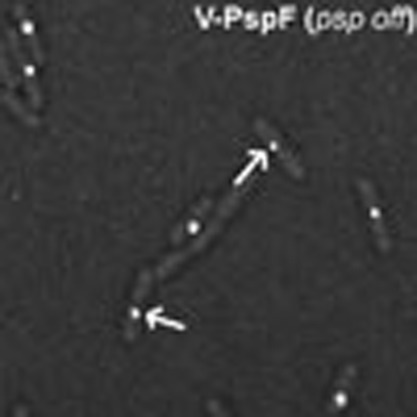

Gene regulation is often achieved at the level of control of transcription initiation where in bacteria sigma (σ) factors have a major role. The bacterial σ^54^ systems are of general importance: they display the key functional properties of many eukaryotic RNA polymerase II promoters that are activated through transcriptional enhancers^1^ and σ^54^ promoters are found in 60% of bacterial species and drive tightly regulated genes used for a wide variety of biological stress-associated functions (for example, pathogenicity, persistence) and biogeochemical cycles^2^.

*In vitro* studies have revealed how homo-hexameric assemblies of specialized AAA+ (ATPases-Associated with diverse cellular Activities) ATPases, bacterial enhancer-binding proteins (bEBPs) bind to the enhancer (upstream activating sequence (UAS)), engage with σ^54^ of the closed promoter complex (RPc) and cause the loss of repressive interactions around a fork junction DNA structure within RPc. Subsequently DNA melting occurs to yield an open promoter complex (RPo) with single-stranded DNA engaged at the active site of RNA polymerase^3^,^4^.

The molecular organization of the enhancing components of the transcription machinery and their coregulators has not been studied *in vivo*. Most ensemble-based assays, *in vivo* or *in vitro,* are limited by averaging that can mask rare states and associated cellular and molecular heterogeneity, so eluding intermediate assemblies and pathway steps. The advent of live-cell single-molecule imaging (SMI) circumvents some of this problems^5^,^6^,^7^,^8^,^9^ and allows detection of complexes refractory to study by conventional approaches. SMI is valuable in providing essential recapitulations of biochemical data, in having the potential to reveal new states of the components including their precise operational stoichiometries, as well as providing insights into how the machineries couple with signalling pathways by revealing their spatio-temporal characteristics. SMI methodology permits a quantitative analysis of functional multi-protein or transient complexes of the bEBP-dependent transcription in the native environment under stress or non-stress conditions.

All cell types have to maintain their membrane integrity for viability. In bacteria a number of membrane-associated stress response systems operate. The widely distributed bEBP-dependent Phage shock protein (Psp) system mounts an adaptation to inner membrane (IM) stress, seen for example in multi-drug resistant persister cells^10^, by repairing the membrane damage and so conserving the proton motive force and energy production^11^,^12^. Many agents induce *psp* expression, and one commonly found inducing condition is the mislocalization of secretins in the IM^11^,^12^. Expression of Psp is σ^54^-dependent and regulated by two-interacting partners: a stress independent low-level expressed bEBP, PspF and its cognate-negative regulator PspA, an IM-associated protein^11^,^12^. A detailed knowledge of PspF and PspA localizations and their self-associations is a key to establishing how the system is controlled and functions *in vivo*. The current models of PspF regulation are fragmentary and based on the ensemble biochemical properties of isolated regulatory components studied in the absence of cell membranes and stress signals, developed in combination with outcomes from invasive cell disruption approaches^12^.

Here by using SMI, PspF fused to fluorescent protein Venus (V-PspF) and a classical inducing agent for Psp in *E. coli*, the secretin pIV of filamentous phage f1 (ref. 12), we characterized the localizations, 2D dynamics and stoichiometry of V-PspF in non-stressed or pIV-stressed live cells. Our data provide evidence for a repressive nucleoid-bound PspF–PspA complex, which dynamically communicates with the IM under non-stress conditions, does not form a stable complex with the IM, and from which PspF dissociates and does not readily rebind if the membrane is stressed. There are two states of association of PspF with the nucleoid depending on non-stress or stress conditions that can be distinguished by diffusion coefficients, each characteristic of a DNA-associated protein complex. The PspF as a single hexamer or subassembly binds a single *psp* promoter at a time, suggesting that *psp* promoters will fire asynchronously. Finally, we revealed the previously uncharacterized cooperative interactions of PspF with the basal transcription complex.

## Results

### Without stress V-PspF is nucleoid-associated and dynamic

**Figure 1:**
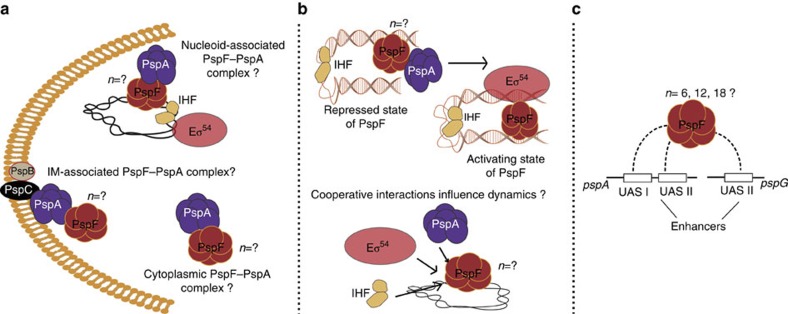
Schematic of propositions tested for PspA-controlled PspF-dependent transcription under stress or non-stress conditions in live *E. coli* cells. (**a**) Localizations of PspF–PspA complex; (**b**) Factors that contribute to different states and dynamics of PspF; (**c**) Stoichiometry of PspF complex (self-assemblies) and the occupancy of *psp* promoters.

**Figure 2:**
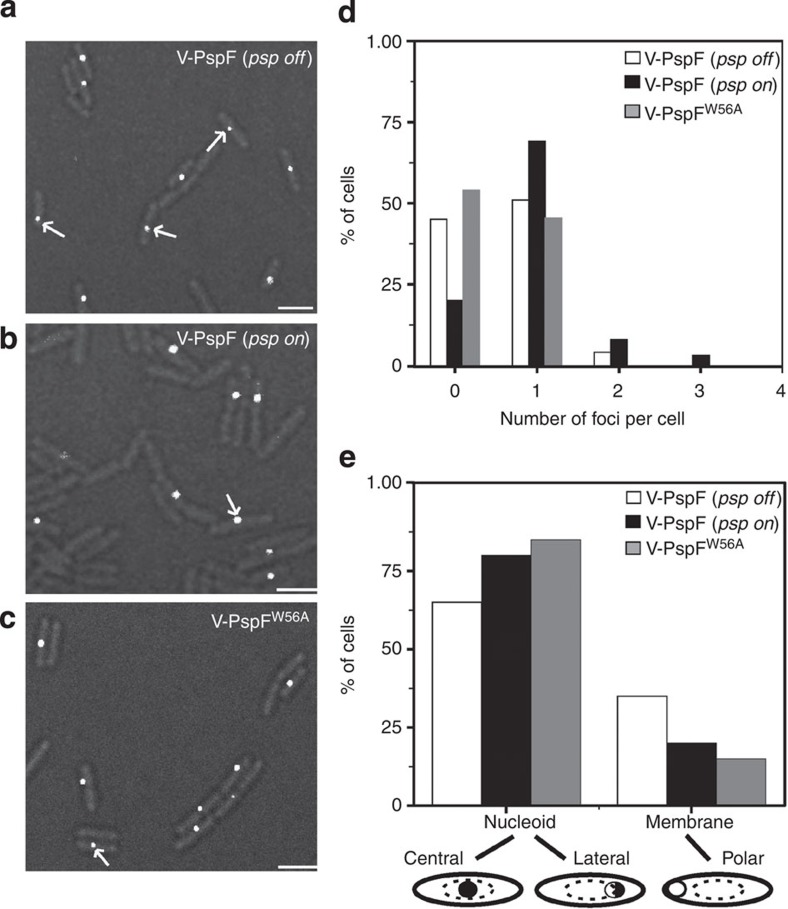
Spatial distributions of V-PspF under non-stress and stress conditions. For SMI, we expressed stable and functional PspF (or its variants) from its native locus as an N-terminal fusion to fast maturing yellow fluorescent protein Venus (V-PspF) (see Supplementary Fig. S1A–C,E,F). *E. coli* MG1655 expressing chromosomal Venus-PspF (V-PspF) under control of its native promoter were imaged under (**a**) non-stress (*psp off*) and (**b**) pIV inducing stress (*psp on*) growth conditions and (**c**) cells expressing Venus-PspF^W56A^ (V-PspF^W56A^) mutant were imaged under non-stress conditions. These proteins are shown as white foci (some marked with white arrow) within the cell (scale bar, 1 μM) in merged images of fluorescent and bright-field images of cells to illustrate of specific localizations. Graphs of (**d**) the number of V-PspF (*n*=314 for non-stress and *n*=197 for stress) or V-PspF^W56A^ (*n*=185) foci per cell where *x* axis represents number of foci and *y* axis represents total percentage of cells, and (**e**) subcellular localizations of the foci on *x* axis (cartoon schematically presents the localization for the V-PspF foci studied) and percentage of cells (*n*=100 for non-stress, *n*=99 for stress and *n*=99 for V-PspF^W56A^) on *y* axis.

PspF is known to bind specifically to the UAS’s of the *E. coli pspA* and *pspG* promoters^13^,^14^,^15^. In non-stressed cells, transcription activation by PspF is repressed by its binding to PspA allowing basal expression of *psp* genes^12^, as shown here for V-PspF (see Supplementary Fig. S1A,E,F). The inhibitory PspF–PspA complex could be cytoplasmic and/or IM bound via PspA (Fig. 1a), thus we examined localizations and diffusion dynamics of V-PspF. In non-stressed (*psp* off) cells (*n*=340), 51% cells had one, 4% two and the remaining 45% of cells were with no discernable foci (wide-field illumination, Fig. 2a). V-PspF foci were predominantly (~60% of stable foci) localized in the nucleoid (central/lateral) with ~40% of relatively transient foci at the polar periphery of the cell (Fig. 2e). The foci in the nucleoid photobleached after 300 ms. However, the polar foci pass into the cytoplasm after 150 ms and do not usually photobleach. When V-PspF foci are lost we cannot discount a dissociation of the V-PspF self-assembly in addition to the free diffusion of a non-DNA-bound self-assembled V-PspF being the mechanism. We used total internal reflection fluorescence (TIRF) microscopy to differentiate between nucleoid and membrane-proximal polar foci; TIRF preferentially images the membrane-proximal region of cells. V-PspF polar membrane-proximal foci were evident in TIRF (Supplementary Fig. S2A), but the central and lateral foci were observed only with wide-field illumination. These findings strongly suggested that V-PspF is predominantly nucleoid-localized with a significant number of foci transiently proximal to the IM in polar regions of the cell (see below). When the plasmid borne non-fluorescent wild-type (WT) PspF was overexpressed in cells expressing chromosomal V-PspF, the V-PspF foci were lost. We infer that the self-association of V-PspF and interactions between V-PspF and promoter DNA and PspA were outcompeted by overproduced WT PspF.

**Figure 3:**
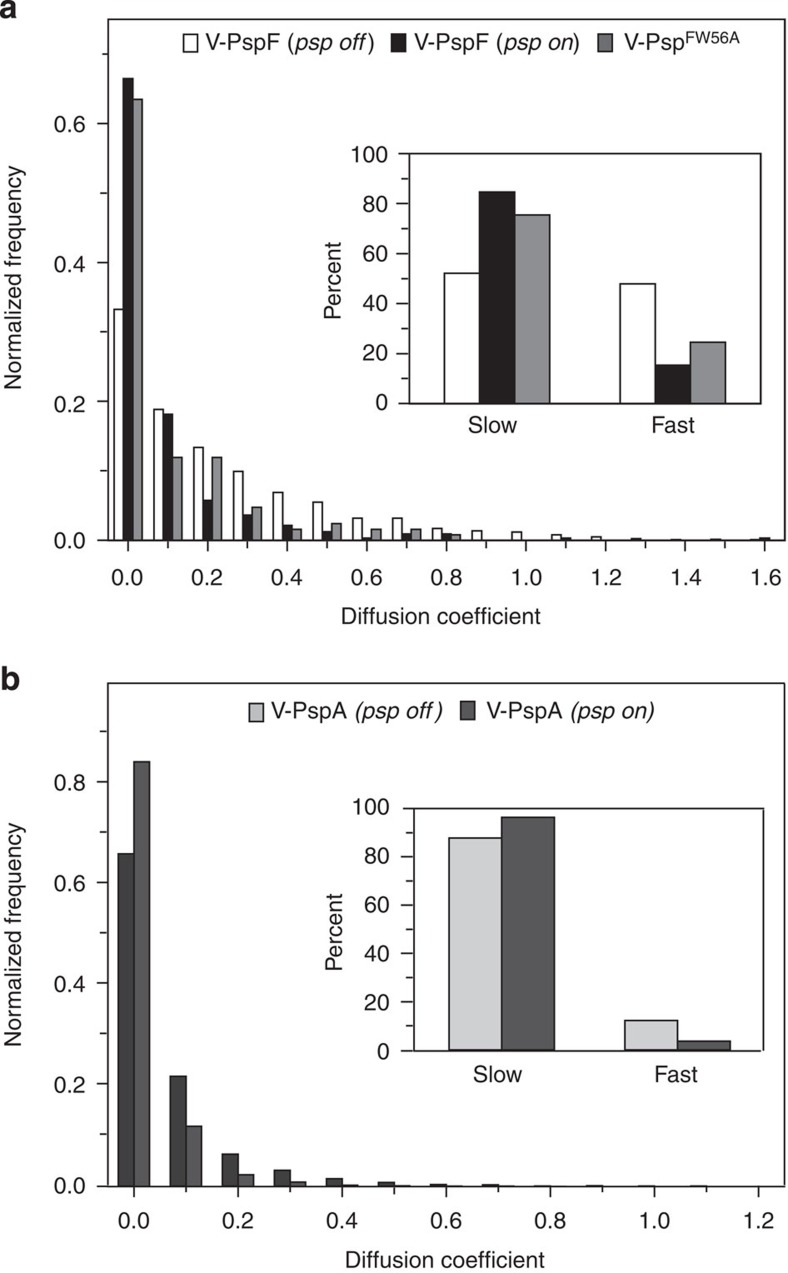
Dynamics of V-PspF is PspA dependent. (**a**) Dynamics of V-PspF under different growth conditions (non-stress, *psp off* (*n*=1423) or pIV-induced stress, *psp on* (*n*=331)) and V-PspF^W56A^ (*n*=126) under non-stress conditions are presented as normalized distribution of the diffusion coefficients (μm^2^ s^−1^) obtained as described in Methods. Inset: the slow/fast foci are classified according to distribution of diffusion coefficients with cutoff at 0–0.15 μm^2^ s^−1^—slow and >0.15 μm^2^ s^−1^—fast. (**b**) The distribution of diffusion coefficients (as in **a**) for V-PspA under non-stress (*psp off*, *n*=7172) and stress (*psp on*, *n*=6485) conditions. Inset: as in **a**.

Next, we established that the dynamics of V-PspF is characteristic of a DNA-bound complex, as defined by apparent diffusion coefficients measured by tracking individual foci (see also Methods). Under non-stress conditions there were near equal numbers of slow (0–0.15 μm^2^ s^−1^) and fast (>0.15 μm^2^ s^−1^) diffusing foci (Fig. 3a inset). The median diffusion coefficient of 0.134 μm^2^ s^−1^ (derived from Fig. 3a) shows that V-PspF dynamics are consistent with nucleoid association, rather than free diffusion (apparent diffusion coefficient >2.5 μm^2^ s^−1^) (refs 16, 17). We assume that V-PspF complexes are predominantly immobilized by DNA or IM association and rarely freely diffuse under non-stress conditions (but do display a range of diffusion dynamics, Fig. 3a). Given the spatial distribution (Fig. 2e), we propose that repressed V-PspF can form dynamic complexes that occasionally commute between the nucleoid and the IM at the cell pole.

### Stress reduces the dynamics of nucleoid-associated V-PspF

In cells stressed by pIV, repression of V-PspF by PspA is lifted (see Supplementary Fig. S1F), as previously shown for the PspF^11^,^12^. Under stress (*psp* on), among 197 cells analysed we observed 69% of cells with single, 8% with 2, 3% with 3 foci and in 20% of the cells no foci were detected (Fig. 2b). The majority (~80%) were stable (35 frames=525 ms) nucleoid-associated foci (not evident using TIRF) with some transient polar membrane-proximal foci (Fig. 2e).

V-PspF in stressed cells showed a significant shift from near equal proportions of slow and fast-diffusing immobilized foci in non-stress (median diffusion coefficient of 0.134 μm^2^ s^−1^) to >80% slow-diffusing foci (median diffusion coefficient of 0.018 μm^2^ s^−1^) (Fig. 3a). The total fluorescence intensity of all V-PspF foci is similar (Supplementary Fig. S2B) as expected for constant levels of V-PspF across growth conditions^12^. The small difference in intensity (about 10%) may reflect its underestimation for fast moving complexes. Overall, the shift in localization and diffusion coefficients of foci in stressed compared with non-stressed cells is likely due to relief of negative control by PspA and the participation of V-PspF in remodelling the RPc at the *psp* promoter(s).

A comparison of wide-field and TIRF-imaged V-PspF provides strong support for its predominant nucleoid rather than stable membrane association. V-PspF did not form a membrane-proximal boundary on summation of images. We conclude that under non-stress conditions V-PspF is nucleoid-associated and dynamic while under stress V-PspF is predominantly within the nucleoid bound to DNA and has reduced dynamics. Quantitative image analysis of DNA-binding fusion proteins, LacI–GFP and σ^54^-YFP (Supplementary Fig. S2C), under our imaging conditions, supports data for V-PspF dynamics. The values for V-PspF diffusion coefficients are in agreement with previously published results for LacI bound to Lac operators^6^,^18^. LacI has diffusion coefficients of 0.046 μm^2^ s^−1^ when scanning for operator binding, 0.4 μm^2^ s^−1^ when bound to non-specific DNA and ~3 μm^2^ s^−1^ when freely diffusing in the cytoplasm.

### Nucleoid association of V-PspF requires specific DNA binding

We studied a variant of V-PspF where the C-terminal DNA-binding domain (containing the helix-turn-helix (HTH) UAS-binding structure)^14^ was removed through the introduction of a stop codon at position 276 (V-PspF_1–275_). It was stably expressed (Supplementary Fig. S1C,E), but did not give any discernible foci. As PspF_1–275_ self-assembles into a hexamer^19^,^20^, we infer the failure to detect foci with V-PspF_1–275_ arises because of the fast diffusion of the soluble non-UAS-bound V-PspF variant, supporting the conclusion that the nucleoid-associated V-PspF foci are specific *psp* UAS DNA-bound complexes.

When V-PspF_1–275_ is expressed from the chromosome PspA and other Psp proteins are not expressed (Supplementary Fig. S3A), as seen for the corresponding chromosomal mutant PspFΔHTH^14^, and so a V-PspF_1–275_–PspA complex is not formed. Accordingly, we did not detect any IM-associated V-PspF_1–275_. As PspF_1–275_ as a hexamer binds six molecules of PspA and is negatively controlled by PspA *in vivo* and *in vitro*^12^,^21^, we reasoned that in principle V-PspF_1–275_ would associate with PspA *in vivo* and might be visible in polar IM-associated regions of the cell. We overexpressed PspA from a plasmid but failed to identify any V-PspF_1–275_ foci (Supplementary Fig. S3B,C). These data imply that in the absence of other Psp proteins, such as the IM sensors PspBC that interact with PspA^22^,^23^, the V-PspF_1–275_-PspA remains soluble (Supplementary Fig. S3E). Alternatively, V-PspF_1–275_ cannot associate with PspA and/or IM-bound PspA *in vivo*. However, the foci of V-PspF_1–275_ are observed at the cells poles when PspBC are coexpressed with PspA (Supplementary Fig. S3D), suggesting assembly of V-PspF_1–275_ occurs *in vivo*, leading to V-PspF_1–275_-PspA interactions and recruitment at the pole via PspBC (Supplementary Fig. S3E).

### Dynamics of V-PspF is PspA-dependent

We further studied the influence of PspA on V-PspF by imaging V-PspF^W56A^, a variant of PspF with a single-amino-acid substitution, which escapes negative control by PspA through abolished binding of PspA to PspF^21^ (see also Supplementary Fig. S1B,E,F). Properties of foci of V-PspF^W56A^ under non-stress conditions (Fig. 2c–f) closely resembled those of WT V-PspF seen in stressed cells (Fig. 2b) in having the following characteristics: most cells had the less dynamic foci in the centre of the nucleoid and their diffusion coefficients (>75% slow-diffusing foci with a median diffusion coefficient of 0.017 μm^2^ s^−1^, Fig. 3a) are similar to those of stressed cells. We infer that the less dynamic central foci are most commonly associated with the transcription activating V-PspF^W56A^ complexes or with the V-PspF complexes under stress, when PspA is not negatively regulating V-PspF. These findings also suggest that the faster dynamics (loss of DNA-binding) of V-PspF and/or any localization of PspF in close proximity with the IM seen under non-stress conditions could be governed by PspA through formation of the PspF–PspA inhibitory complex (Fig. 1a).

Previously, hexameric eGFP–PspA complexes were observed at the IM of the cell poles^24^,^25^ and the effector eGFP–PspA complexes were found to localize in lateral IM regions with MreB-dependent dynamics^24^. To complement V-PspF analyses, we studied the localization and dynamics of V-PspA (Supplementary Fig. S1D,G) under non-stress and stress conditions and correlated outcomes with V-PspF data. Wide-field and TIRF microscopy showed that under non-stress conditions V-PspA foci were mainly localized at the IM polar regions (PspA—regulatory function) with a minority of V-PspA foci in the IM lateral and in nucleoid central regions (Supplementary Fig. S4A,C,D). The diffusion coefficient distributions for V-PspA gave ~90% slow and ~10% fast-diffusing foci with a median diffusion coefficient of 0.030 μm^2^ s^−1^ (Fig. 3b). Clearly the majority of V-PspA foci display a membrane-dependent slow mobility along with a small population of more dynamic nucleoid-associated complexes, likely to be PspF–PspA inhibitory complexes bound at the nucleoid.

Under stress, V-PspA was predominantly localized as IM-associated lateral foci (PspA—effector function) with less polar foci and a minority of central nucleoid foci (Supplementary Fig. S4B,C,E). The onset of stress, enforces the lateral membrane association of PspA and significantly reduces the apparent diffusion coefficient to 0.008 μm^2^ s^−1^, with >90% of foci slowly diffusing (Fig. 3b, inset). Upon stress, V-PspF and V-PspA localizations and dynamics change, presumably as a consequence of the inferred dissociation of the PspF–PspA complex (in polar membrane region) and the subsequent gene activating (central-nucleoid PspF) or effector functions (lateral membrane PspA) needed under stress. *The pspF* transcription under stress is unchanged while the expression of PspA is elevated 100-fold^15^. The number of V-PspA foci under stress is not similarly increased (Supplementary Fig. S4B,E), probably reflecting PspA increasing its oligomeric state to form the effector 36mer (ref. 21).

### V-PspF is often a single promoter-bound hexamer

**Figure 4:**
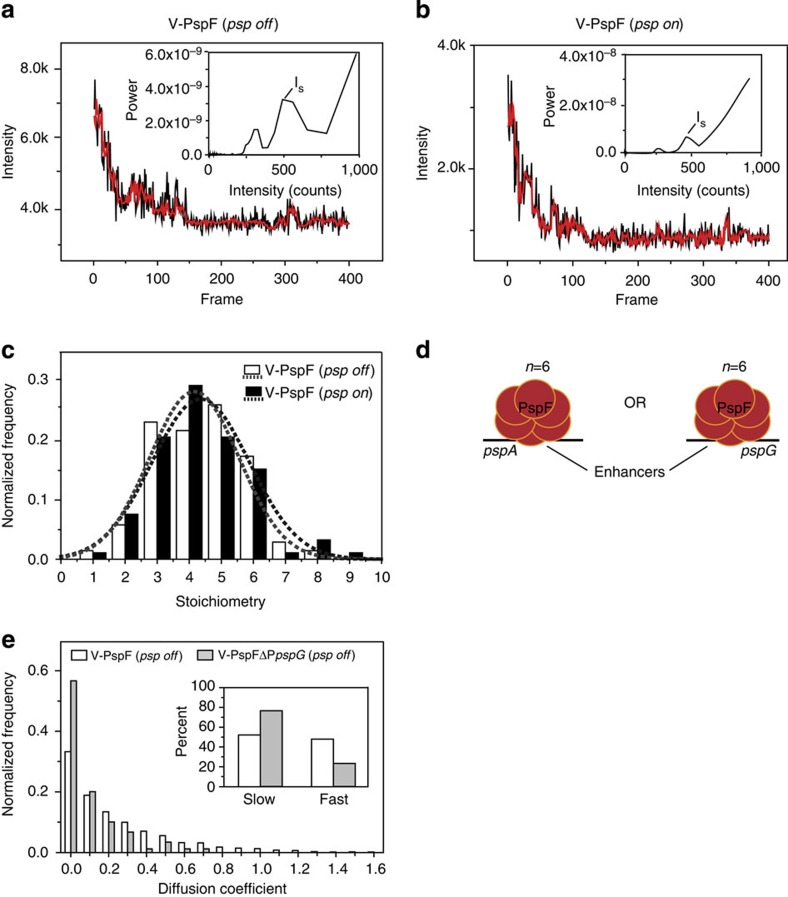
V-PspF stoichiometric analyses and *psp* promoters’-dependent dynamics A representative photobleaching trace of a nucleoid-associated self-assemblies of a PspF complex under (**a**) non-stress (*psp off*, *n*=69) and (**b**) stress (*psp on*, *n*=92) growth conditions and the corresponding filtered trace (in red) based on the Chung–Kennedy algorithm. The insets present the Fourier spectrum of the corresponding pairwise difference distribution curve giving the *I*_s_ (step size); the stoichiometries are calculated using *I*_i_−*I*_f_/*I*_s_ (difference of the initial intensity-*I*_i_ and the final intensity*I*_f_ from the raw data divided by the step size-*I*_s_). (**c**) The distribution of stoichiometries and Gaussian-fit curves calculated from data obtained for V-PspF under non-stress (*psp off*) and stress (*psp on*) conditions. (**d**) A schematic illustration of V-PspF hexamer binding a single *psp* enhancer at a time per cell. (**e**) Normalized distribution of diffusion coefficients (μm^2^ s^−1^) representing dynamics of V-PspF in either non-stressed WT cells (white, *n*=1423) or cells lacking the *pspG* promoter (ΔP*pspG*, grey, *n*=90). Inset: the percentage of foci with slow (0–0.15 μm^2^ s^−1^) and fast (>0.15 μm^2^ s^−1^) diffusion as defined in Fig. 3a legend.

Although it has been shown that the PspF AAA+ domain functions as a hexamer *in vitro*, the active self-associated state of native PspF or indeed any other bEBP *in vivo* is unknown. Moreover, whether or not multiple UASs correlate with binding of multiple PspF species *in vivo* (Fig. 1c) has not yet been addressed. Namely, the *pspA* (29.4 centisomes on *E. coli* map; two UAS’s and 100-fold induced^12^) and *pspG* (91.8 centisomes on *E. coli* map; one UAS and 20-fold induced^12^) promoters may not be simultaneously occupied by PspF. For estimations of stoichiometry of V-PspF, we used photobleaching analysis^25^ (see also Methods) of lateral and central-nucleoid-bound foci (the polar foci are transiently observed and were excluded from this analysis). From the distribution of the single-molecule stoichiometry, we found that the peak was around 4 to 5-mers with a sharp decrease after hexamers under non-stress and stress conditions (Fig. 4a–c). Shu *et al.*^26^ report a similar distribution for hexameric oligomerization, and we infer that the V-PspF complex observed in DNA-bound foci is often a hexamer. Lower oligomeric states were found (Fig. 4c) providing evidence for sub-assemblies. Possibly a range of V-PspF assemblies from dimer up to active hexamer can be present *in vivo* when bound to DNA.

If each *psp* UAS binds one PspF complex, then three hexamers of V-PspF might be found (see Fig. 1c). Exclusively, we observed a single V-PspF hexamer in a cell. This implies that two UAS’s in the *pspA* promoter set the affinity rather than the stoichiometry of the PspF self-assembly and that mainly only one enhancer of the two *psp* promoters (either *pspA* promoter or the *pspG* promoter) is occupied by a V-PspF hexamer at any one time (Fig. 4d). Expression of PspF is unchanged by stress and there could be up to 20 hexamers per cell^27^, suggesting a pool of non-UAS-bound PspF molecules (as hexamers or partial assemblies). Introduction of extra copies of plasmid-localized *pspA* or *pspG* promoters showed up to two foci moving faster than nucleoid-associated one(s) (Supplementary Fig. S5A,B). Presumably, as with LacI and its operators^18^, the V-PspF self-assemblies and their chromosomal DNA-binding constants for specific UAS sites (two to three) and non-specific DNA sites (several million) reflects a thermodynamic requirement of 20 hexamers for stable UAS occupancy.

These results and the number of V-PspF foci per cell indicate that the total amount of available V-PspF complexes could be limited and can form 1 and not >2 DNA-bound foci supporting a model in which the *pspA* and *pspG* promoters/enhancers are rarely if ever occupied at the same time by PspF. We propose that the two *psp* promoters are not often if ever activated at the same precise time.

### The *pspA* and *pspG* promoters interact via V-PspF

The potential for movement of PspF between the *pspA* and *pspG* promoters is a key previously unknown feature of the system, necessary for the normal expression of both promoters. As the *psp* UAS’s occupancy is limited by PspF availability, we expect non-simultaneous expression bursts from the *pspA* and *pspG* promoters on the time scale that would reflect the dissociation and physical passage of PspF from one promoter and binding to the other. To investigate the interplay of V-PspF with *pspA* and *pspG* promoters (including enhancers), we constructed a strain lacking the *pspG* promoter (ΔP*pspG*) and expressing chromosomal V-PspF. Without stress, the spatial organization of V-PspF in ΔP*pspG* was similar to WT P*pspG* although the V-PspF diffusion coefficients were reduced in a ΔP*pspG* mutant (Fig. 4e). We found >70% slow-diffusing V-PspF foci with the median diffusion coefficient of 0.030 μm^2^ s^−1^ (Fig. 4e) somewhat resembling dynamics of V-PspF under stress. In agreement, the basal level transcription from the *pspA* promoter was >3-fold increased in ΔP_*pspG*_ in comparison with WT (Supplementary Fig. S5C). No differences in expression levels were found between WT and ΔP_*pspG*_ under stress.

Thus, removing the *pspG* promoter leads to an imbalanced control of the *pspA* promoter under non-inducing conditions, and impacts on the PspF dynamics with DNA. As the *pspG* gene knock-out (with *pspG* promoter intact) does not impact the PspA expression^15^, it is clear that the *pspA* and *pspG* promoters do functionally interact at the level of PspF.

### The σ^54^ transcription machinery imparts dynamic of V-PspF

Characterizing the engagement of the factors impacting on the activators of the σ^54^ system is especially important, as the activator driven isomerization step for making RPo limits the overall rate of transcription initiation. Thus factors (for example, PspA) that alter the ATPase activity of the activator and/or the probability of contact between enhancer-bound activator and the promoter-bound σ^54^-RNA polymerase (for example, integration host factor (IHF), by bending DNA and facilitating binding of PspF) (see Fig. 1b) can disrupt the control of σ^54^ promoters^4^,^28^.

**Figure 5:**
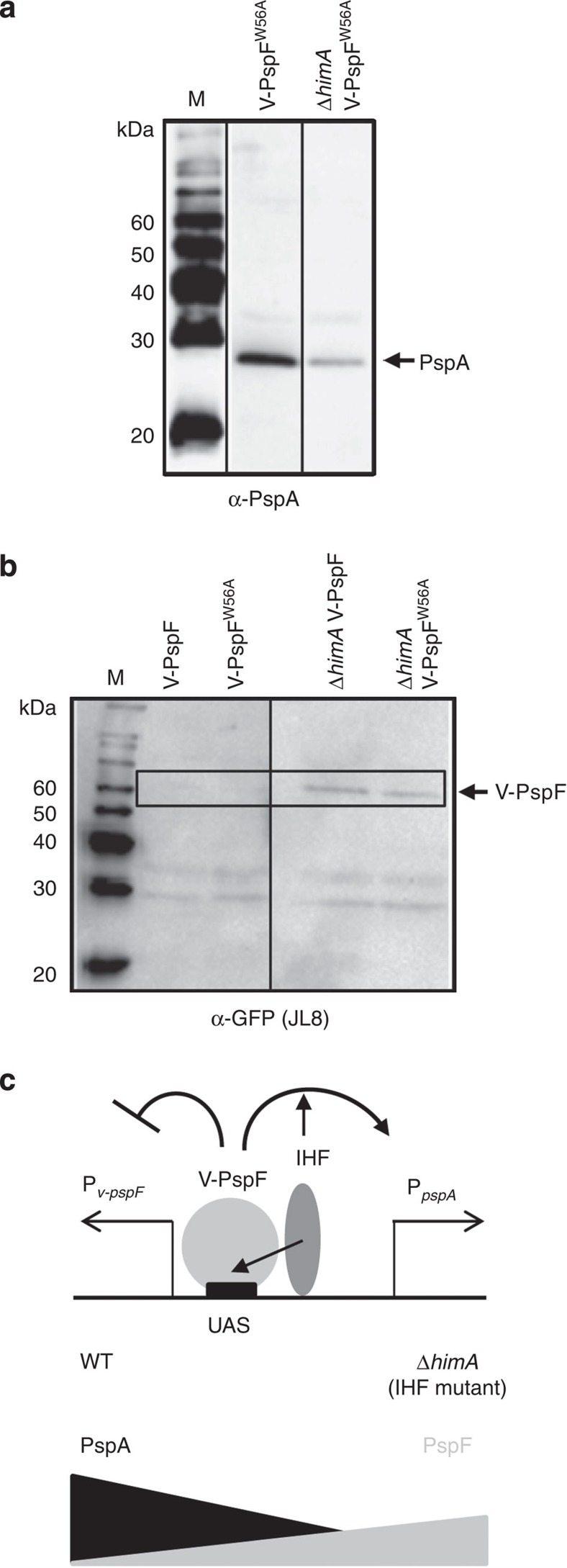
The IHF controls V-PspF and V-PspF^W56A^ binding to the shared regulatory regions of *pspF* and *pspA* promoters. (**a**) Western blot was performed to show the induction of PspA expression using PspA antibodies (α-PspA) in WT or Δ*himA* (IHF mutant) cells expressing V-PspF^W56A^ (no interactions with PspA). (**b**) The expression levels of V-PspF and V-PspF^W56A^ in WT or Δ*himA* cells were determined using western blot and Venus-specific green fluorescent protein (GFP) antibodies (JL-8). (**c**) Schematic presentation of transcription control of *pspF* and *pspA* promoters by V-PspF and IHF. In a WT cells, the IHF assists (full headed small arrows) binding of V-PspF to the UAS in *pspA* (P_*pspA*_) and *pspF* (P_*v-pspF*_) promoter regulatory region and activation of σ^54^-driven *pspA* transcription, and negative control (footed arrow) of σ^70^-driven *v-pspF* transcription. In a Δ*himA* cells (IHF mutant), these controls are diminished leading to decreased activation of PspA (see (**a**) and increased expression of V-PspF (see (**b**))) in agreement with previously published results for native PspF^12^.

To investigate PspF–DNA interactions that may impact on RPc *in vivo*, we characterized V-PspF and V-PspF^W56A^ expression, function, localization and dynamics in cells lacking the WT IHF (Δ*himA*), which assists binding of PspF to UAS DNA and facilitates PspF–RPc interactions through DNA-looping^12^. We first showed that IHF controls the expression of V-PspF and expression and function of V-PspF^W56A^ consistent with our evidence that these proteins are bound to specific DNA UAS’s (Fig. 5): V-PspF^W56A^ stimulates enhancer-dependent transcription at the *pspA* promoter in an IHF-dependent manner and V-PspF and V-PspF^W56A^ negatively regulate their own expression in an IHF-dependent manner, upon binding of V-PspF or V-PspF^W56A^ to the *pspA* enhancer.

**Figure 6:**
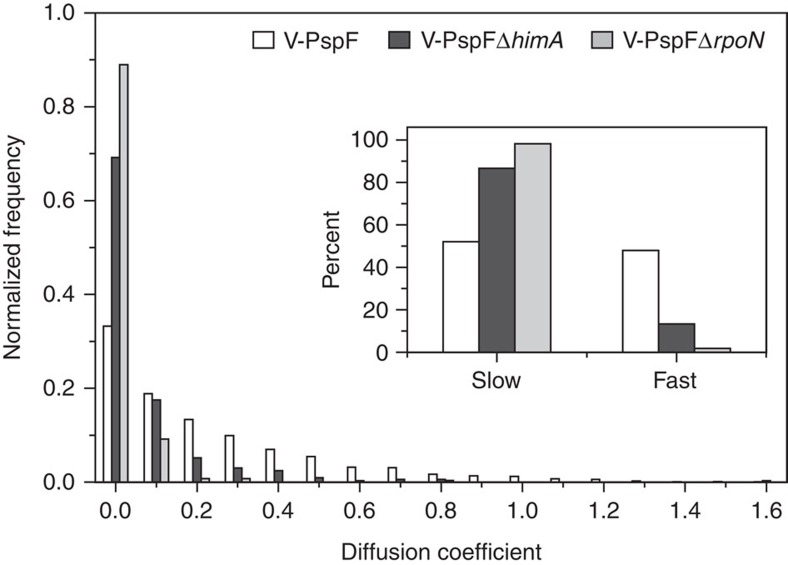
The σ^54^ transcription machinery affects dynamics of bEBP PspF. Normalized distribution of diffusion coefficients (μm^2^ s^−1^) representing dynamics of V-PspF in either Δ*himA* (IHF mutant) cells (white, *n*=331) or Δ*rpoN* (no σ^54^) (black, *n*=271) cells. Inset: the percentage of foci with slow (0–0.15 μm^2^ s^−1^) and fast (>0.15 μm^2^ s^−1^) diffusion as defined in Fig. 3a legend.

Without stress, the loss of WT IHF caused an increased number of V-PspF foci (up to 4 per cell) in close proximity to the membrane (observed also by TIRF imaging) (Supplementary Fig. S6A,D,E) and V-PspF exhibited 10-fold reduction in dynamics (compared with WT) with >80% slow-diffusing foci with a median diffusion coefficient of 0.015 μm^2^ s^−1^ (Fig. 6). The results with V-PspF^W56A^ showed that both localization (Supplementary Fig. S6B, F) and dynamics (Supplementary Fig. S6H) in a Δ*himA* are similar to V-PspF and so are independent of PspA.

The remodelling target of PspF is σ^54^ in RPc^29^, and so we characterized V-PspF using cells lacking σ^54^ (Δ*rpoN*). In non-stressed Δ*rpoN* cells the V-PspF was mainly nucleoid centrally and laterally localized with very few polar membrane foci (Supplementary Fig. S6C,D,G). Lack of σ^54^ and hence RPc and PspA reduced the dynamics of V-PspF, exhibiting 95% of slow-diffusing foci with the median diffusion coefficient of 0.008 μm^2^ s^−1^ (Fig. 6). Overproduction of plasmid borne PspA yielded additional short lived (survival time 105 ms) membrane V-PspF foci and increased V-PspF dynamics in σ^54^ mutant with a median diffusion coefficient of 0.014 μm^2^ s^−1^ (Supplementary Fig. S6I).

Clearly the σ^54^ transcription machinery affects subcellular localizations and dynamics of V-PspF as a direct consequence of protein–DNA and protein–protein interactions, and possibly through modifying the local DNA architecture.

## Discussion

We show that control of the pervasive Psp stress response operates through a post-DNA-binding repression of PspF, a specialized bEBP. It appears that often the repressive complexes of V-PspF–PspA reside in the nucleoid (Supplementary Fig. S7). Such complexes are dynamic as highlighted in their broad distribution of diffusion coefficients, indicating a capacity to communicate with the IM (Fig. 3). The proposed occasional excursions of repressive complexes away from the nucleoid towards the IM will allow the system to switch from the repressed ‘off’ state to the gene activating ‘on’ state upon stress. The V-PspF movement from the nucleoid to the IM is PspA-dependent (Supplementary Fig. S7). We do not exclude the possibility that following a dissociation of the nucleoid V-PspF–PspA complex there may be an IM-based reassembly of the V-PspF–PspA complex. In the gene activating states, V-PspF were more stably bound to the central-nucleoid position with a seven-times reduction in dynamics (Fig. 3), reflecting a loss of PspA binding to PspF, action of IHF and an engagement with the closed RPc or/and open RPo promoter complex(es) (see below). The reduced dynamics of a regulator whose movement could be obstructed by interaction with other protein(s) stably bound to an adjacent-specific DNA sequence is in agreement with results recently published for LacI DNA sliding, which can be reduced by other proteins bound to DNA near the operator^30^.

Our analysis of the subunit stoichiometry of V-PspF indicates the presence of hexameric assemblies, and the distributions of numbers of Venus molecules per foci under both non-stress and stress conditions suggest some assemblies containing less than six subunits can be found (Fig. 4a–c). We infer the hexameric assemblies are active for stimulating transcription, the smaller assemblies may be intermediates en route to forming mature hexamers and are likely inactive for the ATPase activity needed to stimulate RPo formation. The stoichiometry of the repressed and the activating ATP hydrolyzing states of PspF are the same implying no regulation by changes in the oligomeric state of this bEBP *in vivo.* Significantly, we obtained data strongly suggesting that PspF stably binds only one promoter at a time, and so would need to bind two target promoters on average in turn for a balanced control in their activation (Fig. 4d). This promoter order strategy might be a general feature of regulons controlled by a limiting amount of a regulator and may be commonly observed when a regulon expands. Unless compensated by upregulation of the transcription factor involved, single promoter occupancies may lead to increases in heterogeneity of gene expression between cells under stress. In general, the changes in sequence or number of the *cis* regulatory sequences may drive the microevolution of chromosomes and could have a central role in establishing developmental differences between bacterial strains and so, as already proposed by others^31^,^32^,^33^, lead towards differentiation of species.

The diffusion coefficients and localization of V-PspF are highly dependent on its cognate-negative regulator PspA. The interactions with PspA seem to control the membrane association and dynamics of PspF complexes at the level of inhibitory complex assemblies. The diffusion coefficients and localization of V-PspF are further influenced by RPc and IHF, suggesting an intricate balance between nucleoid and membrane associations of PspF underpinned by layers of cooperativity between the Psp components and the local architectural element, IHF. These interactions had escaped standard biochemical detection in *in vitro* assays.

Our findings provide a quantitative understanding of the eukaryotic-like enhancer-dependent σ^54^ promoter control, establishing the vital role of communication between nucleoid and membrane, in which DNA and IM-interacting complexes communicate information about membrane damage, leading to adaptation through transcription control (Supplementary Fig. S7). The control scheme includes non-simultaneous promoter use with direct implications in establishing the Psp stress response in biofilms, virulence settings and the multi-drug resistance of persister cells^10^,^11^,^12^,^34^.

## Methods

### Bacterial strains and growth conditions

Bacterial strains used in this study are listed in Supplementary Table S1. Strains MG1655 V-PspF Δ*rpoN*, MG1655 V-pspF Δ*himA*, MG1655 V-PspF^W56A^ Δ*himA*, DY226 λV-PspA, MVA127 and MVA131 were constructed using P1_vir_ transduction (see Supplementary Table S1). The constructions of strains MG1655 V-PspF, MG1655 V-PspF^W56A^, MG1655 V-PspFΔHTH and SA1943 λV-PspA expressing chromosomal PspF/derivatives or PspA fused to fast-maturing fluorescent protein Venus^35^, and MVA129 lacking the *pspG* regulatory region are described in detail in Supplementary Methods; see also Supplementary Table S1. The strains were grown under micro aerobic conditions in Luria-Bertani (LB) broth or on LB agar plates at 37 °C (ref. 36). The cultures for microscopy and western blots were grown at 30 °C in N^−^C^−^ minimal media supplemented with 0.4% glucose as carbon source, 10 mM NH_4_Cl as nitrogen source and trace elements. The pIV secretin was constitutively expressed from pGJ4. The PspA and PspF proteins were expressed from pBAD *ara* promoter in pPB10 and pPB8-WT, respectively, in the presence of 0.1% final arabinose (Ara). The expression of PspBC sensors from plasmid pAJM3 is induced by 0.02% Ara. The expression of PspA from pPB9 plasmid is induced by 0.1 mM isopropyl-beta-D-thiogalactopyranoside. When required, the bacterial cultures and plates were supplemented with antibiotics at following concentrations: ampicillin, 40 or 100 μg ml^−1^; kanamycin, 25 or 50 μg ml^−1^; tetracycline, 10 μg ml^−1^; chloramphenicol, 30 μg ml^−1^; spectinomycin 100 μg ml^−1^. Transformations and P1_vir_ transductions were performed as described^36^ (see Supplementary Table S1).

### Western blotting

Total cell extracts were from cells grown in minimal medium, harvested at mid-exponential phase (normalized according to OD_600_), resuspended in a mix of 30 μl 4% SDS and 30 μl Laemmli Buffer (Sigma) and boiled at 100 °C. The samples were separated by SDS–polyacrylamide gel electrophoresis and transferred on to a polyvinylidene difluoride membrane using a semidry transblot system (Bio-Rad). The western blotting was performed on a Bench Pro 4100 Card processing station Invitrogen. Antibodies used were JL-8 Living Colours (Clonetech) against Venus (1:1000 or 1:5000), PspA antibodies (1:1000), and pIV (1:10000). The proteins were detected using enhanced chemiluminescent (ECL) plus western blotting detection kit. Images were digitally acquired using Bio-Rad GelDoc and ChemiDoc systems with Image Lab software and analysed using Adobe Photoshop CS3.

### β-Galactosidase assays

The activity from the chromosomal φ(*pspA-lacZ*) transcriptional fusion was assayed to estimate the level of *psp* expression under non-stress and stress growth conditions. The overnight cultures were grown at 37 °C in LB broth and cells were diluted 100-fold and grown to mid-exponential phase for β-galactosidase assay^36^. The assay was performed in triplicate for two independent biological samples, and data are the average values with s.d. values.

### Microscopy and data analyses

The bacterial cells expressing V-PspF/derivatives (see Supplementary Fig. S1A–C,E,F) and V-PspA fusions (see Supplementary Fig. S1D,G) in different backgrounds were grown at 30 °C in N^−^C^−^ minimal media supplemented with 0.4% glucose as carbon source, 10 mM NH_4_Cl as nitrogen source and trace elements. The cells were immobilized on 1% agarose pads set on a glass slide surface as described^24^. Two imaging systems were used: The first system was a custom-built inverted epifluorescence/TIRF microscope based on a Nikon TE2000 optical microscope, a tuneable argon ion laser and an electron-multiplying charge-coupled device (EMCCD) camera for live-cell single-molecule fluorescence imaging. The sample was excited with the 514 nm line of the argon ion laser (35LAP321-230, Melles Griot). An Apo TIRF numerical aperture 1.49 oil immersion objective (Nikon) and a dichroic filter set (zt514 TIRF, Chroma Technology) were used for diffraction limited imaging^25^. Fluorescence images were recorded by a Cool-View EM1000 EMCCD camera (Photonic Sciences). The laser power was 5 mW, exposure time 15 milliseconds (ms) and captured frame sequences with 2 × 2 binning at a frame interval of 29 ms. The second system was Deltavision OMX V3 (Applied Precision, Washington) with 3 ms exposure time at a frame interval of 44 ms and was mainly used for photobleaching experiments using 10% laser power at 514 nm. The images were analysed using image analysis software ImageJ ( http://www.rsbweb.nih.gov/ij/) and FiJi. The diffusion analysis was as described^37^ using Matlab (Mathwork) scripts (see Supplementary Methods and Supplementary Fig. S8). Photobleaching of individual V-PspF foci from 69-non-stressed and 92-stressed cells was determined using an edge preserving algorithm combined with Fourier spectral analysis^25^ (see details in Supplementary Methods); data were also analysed using manual counting methods (see details in Supplementary Methods and Supplementary Fig. S9). Fluorophores in the dark states or already bleached before measurement were beyond quantification by the chosen method.

## Author contributions

Conceived and designed experiments: M.B., G.J., L.Y., P.M. Performed the experiments: P.M., G.J., T.L., A.B., C.E. Analysed the data: P.M., G.J., A.B., L.Y., M.B. Contributed reagents/materials/analysis tools: A.B., L.Y., M.B. Wrote the paper: G.J., M.B, P.M. Involved in all the experiments: P.M. Involved in SMI microscopy experiments: P.M., A.B., T.L., LY.

## Additional information

**How to cite this article:** Mehta, P. *et al.* Dynamics and stoichiometry of a regulated enhancer-binding protein in live *Escherichia coli* cells. *Nat. Commun.* x:x doi: 10.1038/ncomms2997 (2013).

## Supplementary Material

Supplementary Information Supplementary Figures S1-S9, Supplementary Table S1, Supplementary Methods and Supplementary References
