# Supplementary material for: Dynamics and stoichiometry of a regulated enhancer-binding protein in live Escherichia coli cells
Source: Nat Commun. 2013 Jun 14;4:1997. doi: 10.1038/ncomms2997 (PMC3709507; doi:10.1038/ncomms2997)
Supplement: Supplementary Information — Supplementary Figures S1-S9, Supplementary Table S1, Supplementary Methods and Supplementary References [file ncomms2997-s1.pdf]

Supplementary Information

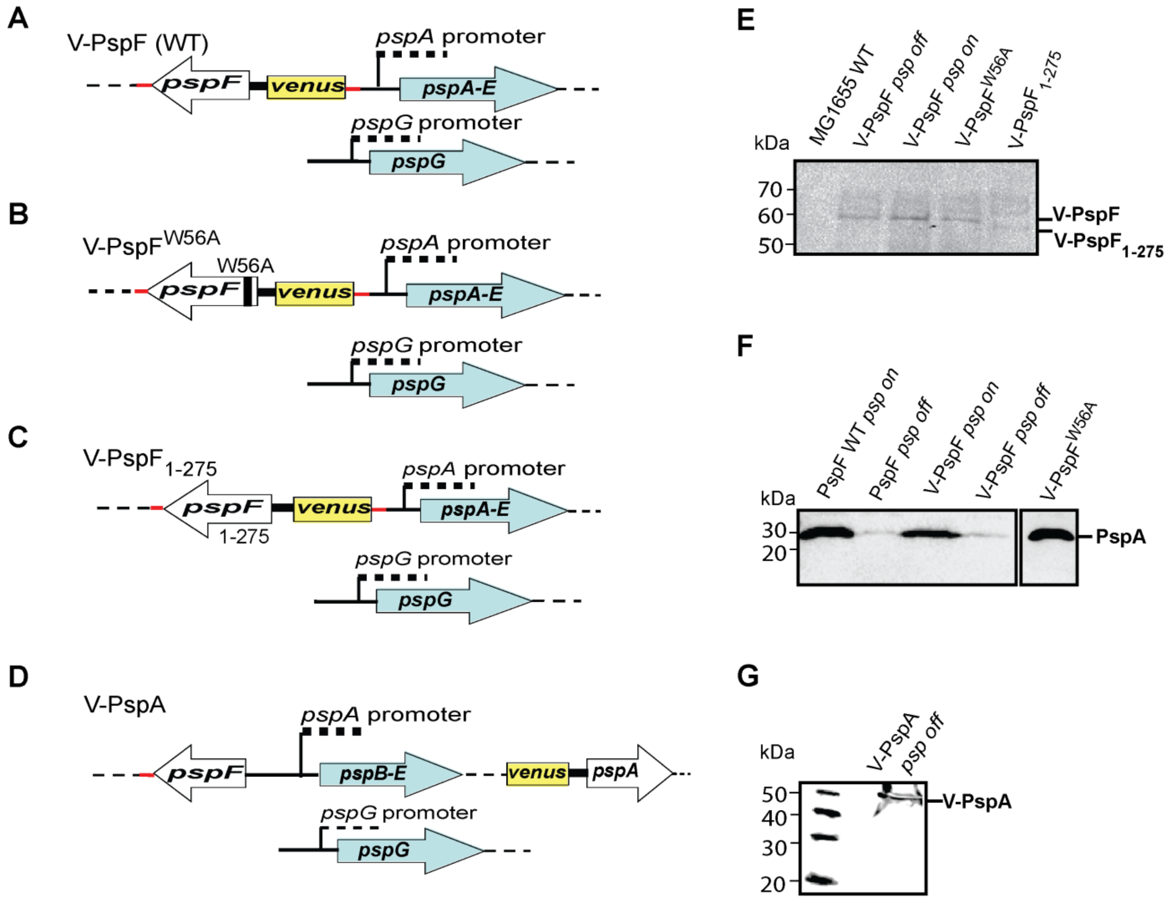

Supplementary Figure S1. The Venus-PspF (V-PspF), its variants and Venus-PspA (V-PspA) fusion protein constructs, their expression, stability and functionality. (*A*-*C*) Schematic organisation of the *psp* genes in strains expressing V-PspF and its variants; the *venus* gene and the linker are fused to the N-terminus of *pspF*, so retaining the other *psp* genes at their native locations. (*A*) V-PspF WT; (*B*) V-PspF^{W56A} mutant, lacking the repressive binding interaction with PspA and (*C*) V-PspF$_{1\text{-}275}$, with a deletion of the DNA binding domain. (*D*) Schematic organisation of the *psp* genes in strains expressing V-PspA; *venus* gene and the linker are fused to the N-terminus of *pspA* under control of native *pspA* promoter with native *pspA* gene deleted. (*E*) Western blot was performed to show the expression level and stability of the V-PspF (non-stress, *psp off*, and stress *psp on*, conditions), V-PspF^{W56A} (~64 kDa) and V-PspF$_{1\text{-}275}$ (*psp off*) (~60 kDa) using Venus specific GFP antibodies (JL-8). (*F*) Western blot was performed to show the functionality of different V-PspF proteins (activation of *pspA* gene) using PspA antibodies; as shown the V-PspF WT fusion behaved as native PspF, supporting a basal level expression of PspA under non-stress conditions which was activated by V-PspF with the onset of stress; transcription of the *psp* genes is constitutively switched on in V-PspF^{W56A}. (*G*) Western blot against Venus specific GFP antibodies (JL-8) was performed to show the expression and stability of the V-PspA (~53 kDa).

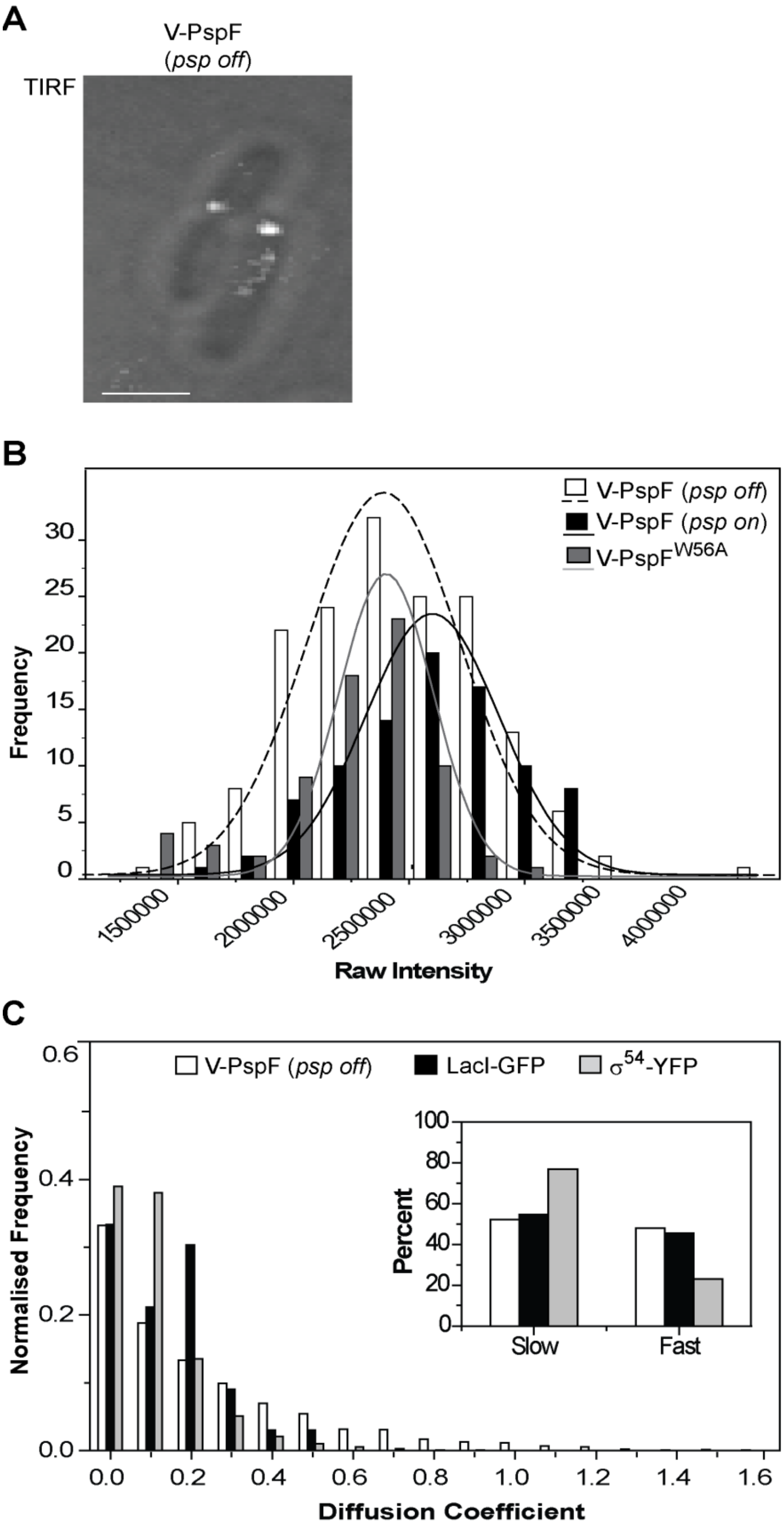

Supplementary Figure S2. Localisation and dynamics of V-PspF. *(A)* Total Internal Reflection Fluorescent (TIRF) microscopy of V-PspF under non-stress growth conditions. To help distinguish between the membrane or soluble/nucleoid associations, the V-PspF was

imaged in a TIRF mode. The merge image of fluorescent and bright field images of V-PspF foci seen at the poles of the cell (scale bar=1μM); this image represents the 5th frame of the 100 frame video sequence, with frame rate of 30 frames/s. The V-PspF foci imaged in the TIRF mode were seen for shorter period of time, 135 ms, as opposed to 300 ms in a Wide Field (WF) mode. There are foci in the middle of the cell visible in wide-field epi-fluorescence that are not visible in TIRF mode. The diffusion coefficients of these foci are similar to those of known DNA binding proteins. These foci are not detectable when a PspF variant which cannot bind DNA is studied. Therefore centrally located V-PspF foci are likely to be nucleoid, target promoter DNA associated PspF. *(B)* The raw intensities of V-PspF (*psp off*/*on*) and V-PspF^{W56A}. The image sequences of 100 frames (1.5 s long) were added together to obtain a summed image, where the intensity for each of the foci was calculated and called the raw intensity. The raw intensity was calculated for 100 foci of V-PspF under non-stress or stress conditions, and for 60 foci of V-PspF^{W56A} under non-stress conditions. The graph presents the distribution of the raw intensity on x-axis against the frequency distribution on the y-axis; V-PspF non-stress (white), V-PspF stress (black) and V-PspF^{W56A} (grey). Gaussian curves show a moderate shift of the raw intensity to the right for V-PspF under stress compared to other two data sets. Immobile complexes were excited in the centre of the focus plane. We estimate that the mean displacement of the complex in non-stress cells is about 125 nm in 30 ms exposure time, suggesting that very mobile complexes could move out of the focal plane within one frame, giving apparent lower intensity. *(C)* Comparisons of apparent diffusion coefficients between V-PspF and other relevant DNA binding proteins. The magnitude of the diffusion coefficient obtained for V-PspF is that of a DNA binding protein as reported in literature for e.g. LacI. In order to confirm these findings we directly determined diffusion coefficients of relevant DNA binding proteins, LacI-GFP expressed from the chromosome in MG1655, and σ^{54} (σ^{54}-YFP expressed from the plasmid pCE1); σ^{54} is direct PspF interacting partner in the RPc and/or RPo. The normalised distribution of apparent diffusion coefficients for LacI-GFP (black, n=66), σ^{54}-YFP (grey, n=12624) and V-PspF (white, n=1423) is shown. The V-PspF foci showed similar dynamics in comparison to LacI-GFP and σ^{54}-YFP foci. LacI-GFP presents an important control to calibrate and establish directly the method we employed to quantify dynamics of V-PspF. The median diffusion coefficients of 0.13 $\mu m^2 s^{-1}$ and 0.071 $\mu m^2 s^{-1}$ for LacI-GFP and σ^{54}-YFP respectively were obtained and are similar to one found for V-PspF, 0.13 $\mu m^2 s^{-1}$. Inset: LacI-GFP and V-PspF has a very similar percentage of slow and fast diffusing foci, while σ^{54}-YFP has more slow diffusing foci.

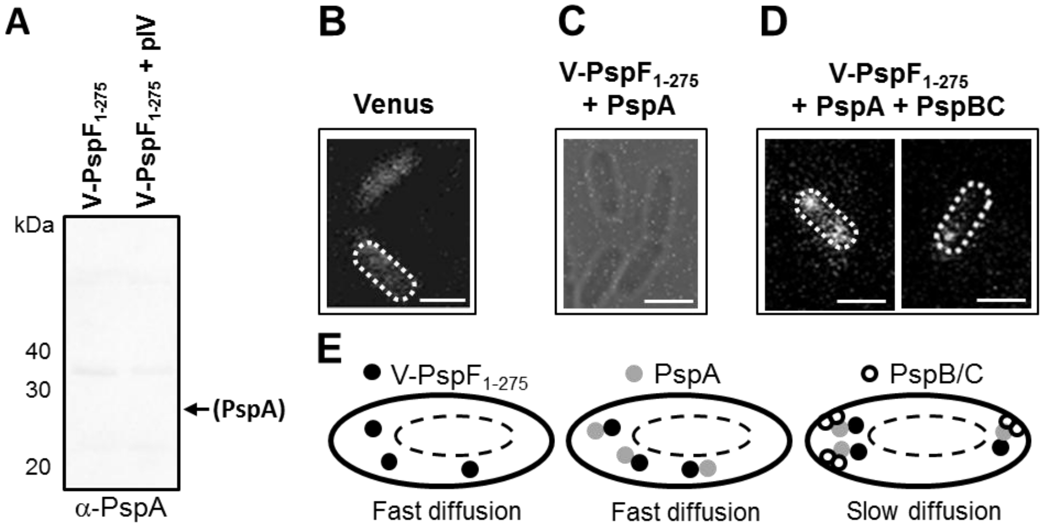

Supplementary Figure S3. PspBC IM sensors recruit the PspA-V-PspF$_{1-275}$ complex in the polar region of the cell. (*A*) Western blot using PspA antibodies (α-PspA) to determine the expression of PspA in MG1655 V-PspFΔHTH cells before or after stress. The chromosomal V-PspF$_{1-275}$ (MG1655 V-PspFΔHTH) do not activate or induce the expression of PspA under non-stress or stress (+pIV, pGJ4) conditions, respectively. (*B*) The fluorescent wide field images of MG1655 cells expressing Venus alone from the pCS2 plasmid. (*C*, *D*) The merge fluorescent and the bright field images of V-PspF$_{1-275}$ foci (MG1655 V-PspFΔHTH) in wide field mode of cells over-expressing *in trans* (*C*) PspA (pPB9) or (*D*) PspA (pPB9) and PspBC (pAJM3). *B-D*, scale bar=1μM. (*E*) Schematic presentation of cells expressing V-PspF$_{1-275}$ (black circle) which does not bind the DNA/nucleoid (dashed line) or IM (full line) and cannot be visualised because it is fast diffusing in the cytoplasm. The chromosomal physiological expression of V-PspF$_{1-275}$ that lack DNA-binding motif is not sufficient to activate *pspA* (and *pspG*) promoter [(see (*A*)]. The PspA (grey circle) expressed *in trans* from the plasmid is able to bind V-PspF$_{1-275}$ but the complex still remains in the cytoplasm. The co-expressions of PspA and PspBC (open circle) enable visualisation of the V-PspF$_{1-275}$ because the PspBC sensors recruit the PspA-V-PspF$_{1-275}$ complex onto the IM in polar region of the cell.

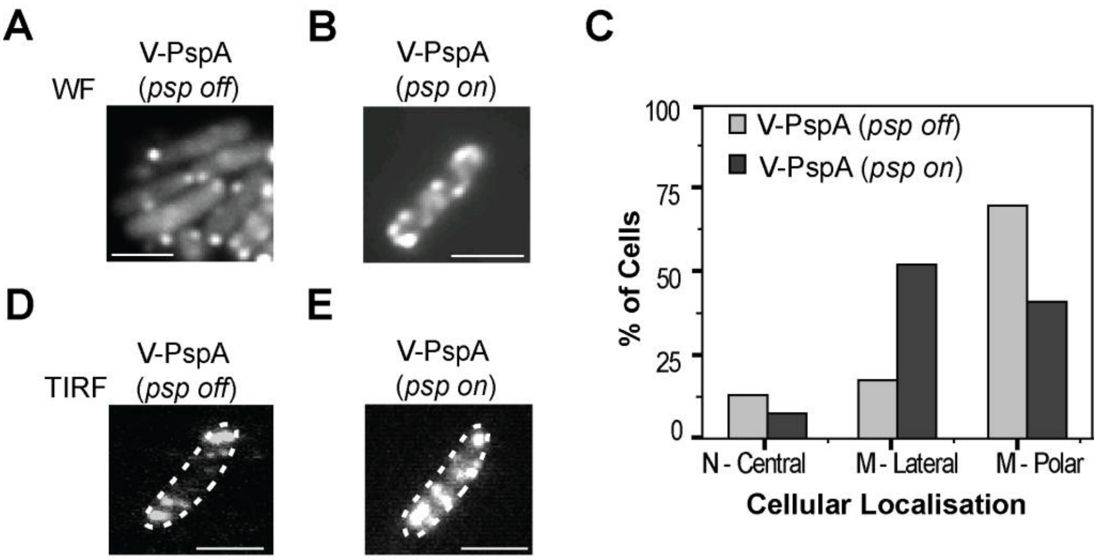

Supplementary Figure S4. Localisations of V-PspA under non-stress and stress conditions. Images of MG1655Δ*pspA* cells expressing chromosomal (inserted into *attB* site) Venus fusion of PspA (V-PspA) under control of *pspA* promoter with native *pspA* deleted and native *pspF pspBCDE* and *pspG* intact: (*A*) images of cells under non-stress (*psp off*) conditions displaying the V-PspA foci at predominantly polar localisations; (*B*) V-PspA foci localisations under pIV inducing stress conditions (*psp on*) show increase in the number of foci, with polar and lateral foci; (*C*) The profiles of V-PspA localisations under *psp off* (light grey, n=115) and *psp on* (dark grey, n=135) conditions; N-Central - nucleoid central, M-Lateral - membrane lateral, M-Polar – membrane polar. TIRF image of V-PspA expressed under (*D*) non-stress (*psp off*) and (*E*) stress (*psp on*) conditions in MG1655Δ*pspA* cells (with defined cell boundary); the V-PspA polar and lateral foci observed confirms the membrane (proximity) association. Images *A*, *B*, *D* and *E* (scale bar=1μM).

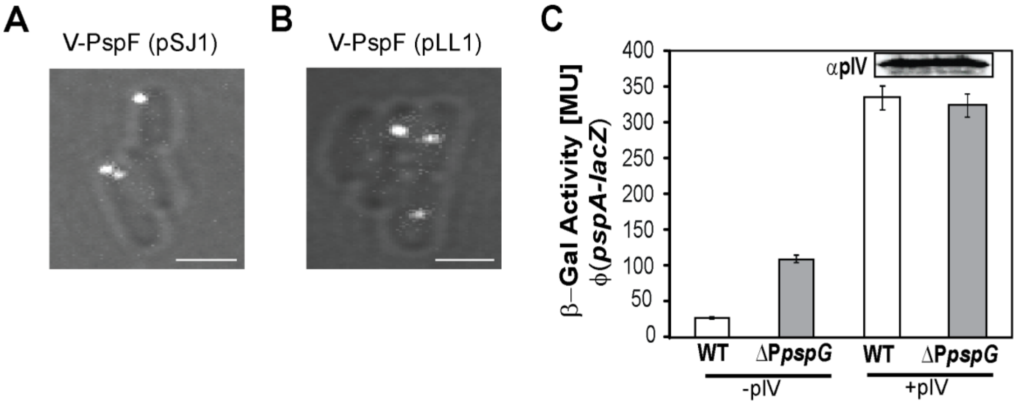

Supplementary Figure S5. V-PspF concentration is limiting and leads to an imbalanced control of the *pspA* promoter under non-inducing conditions. *(A, B)* Adding extra copies of *psp* promoters. The MG1655 strain (*psp off*) expressing V-PspF was transformed with the plasmids carrying extra copies of the (*A*) *pspA* promoter (pSJ1; ~50 copies per cell) or (*B*) *pspG* promoter (pLL1; ~5 copies per cell) to examine their effects on the localisations and/or total number of V-PspF foci observed; The addition of extra copies of *pspA* promoter did not lead to an increase in the number of V-PspF foci but the foci were found to be more polar (extra-chromosomal) with up to two foci observed; the addition of *pspG* promoter did not show an obvious change in the number or localisation of the V-PspF foci. The images *A* and *B*, scale bar=1μM. *(C)* The β-Galactosidase (β-Gal) activity (MU, Miller Units) was measured using chromosomal transcription fusion ϕ(*pspA-lacZ)* in MG1655 wild type (WT, white) or MG1655 strain lacking *pspG* promoter region (ΔP*pspG*, grey) under non-stress (-pIV) or pIV inducing stress (+pIV) conditions. The results show the increased basal level expression of *pspA* in the absence of *pspG* promoter. The assay was performed in triplicate for two independent biological samples and data are the mean values with SD error bars.

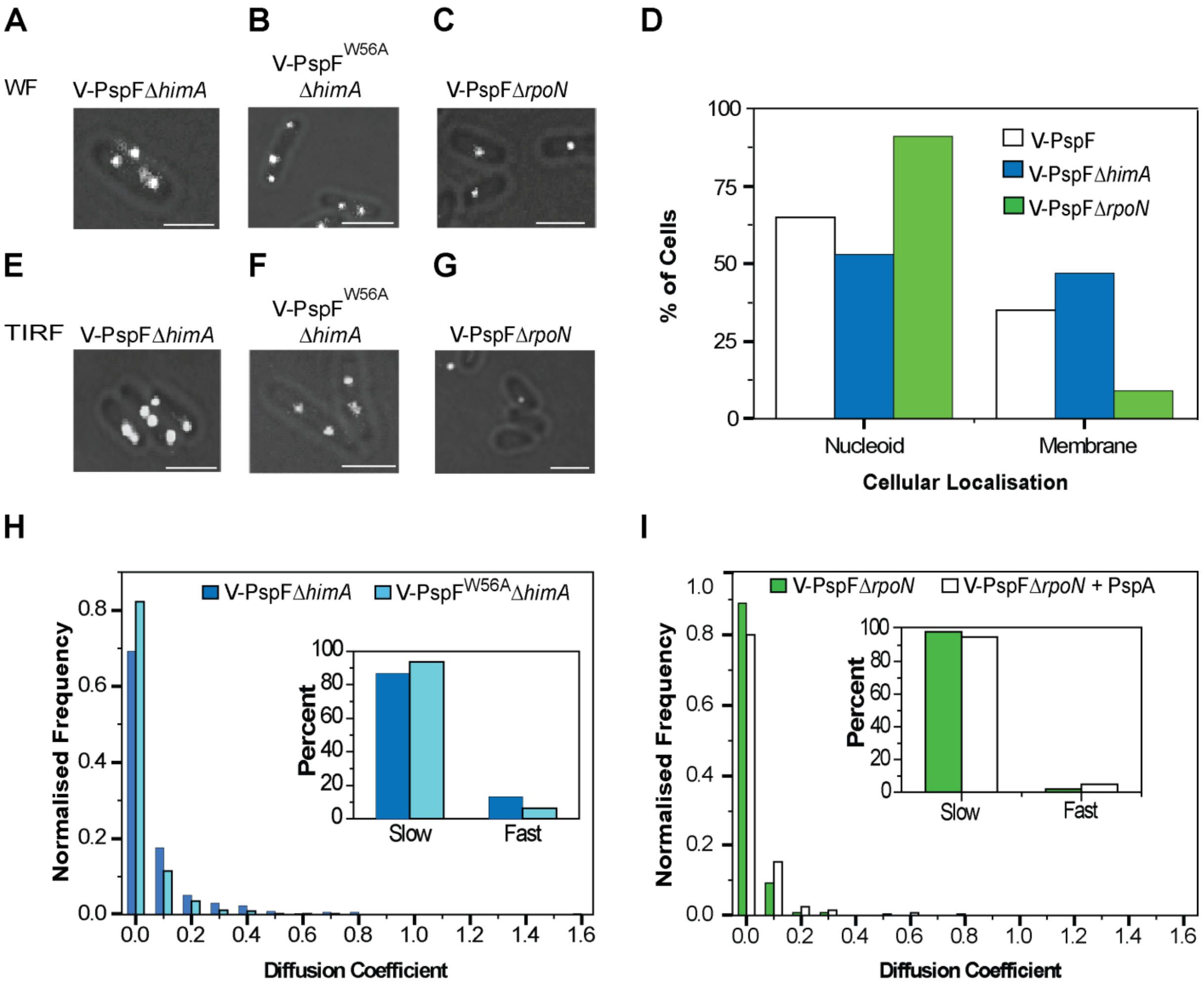

Supplementary Figure S6. The localisations and dynamics of V-PspF in the absence of IHF or σ^{54} under non-stress conditions. The merge fluorescent and bright field images (scale bar=1μM) of V-PspF foci comparing (*A*-*C*) Wide Field (WF) and (*E*-*G*) TIRF modes: WF/TIRF - (*A*)/(*E*) V-PspF foci in Δ*himA* (IHF mutant) cells localise predominantly at polar and lateral membrane proximal positions; (*B*)/(*F*) V-PspF^{W56A} foci in Δ*himA* localise similarly to V-PspF; (*C*)/(*G*) V-PspF foci in Δ*rpoN* (no σ^{54}) are predominantly localised as central nucleoid foci; (*D*) the summary of V-PspF localisations in wild type (WT, white, n=100), IHF mutant (Δ*himA,* blue, n=57) and σ^{54} mutant (Δ*rpoN*, green, n=54) backgrounds. *(H)* The dynamics of V-PspF (dark blue, n=331) and V-PspF^{W56A} (light blue, n=445) in *ΔhimA* strain represented by normalised distribution of diffusion coefficients showed very similar distribution for the two proteins. Inset: the slow/fast foci are classified according to distribution of diffusion coefficients with cut off at 0-0.15 $\mu m^2 s^{-1}$ - slow and >0.15 $\mu m^2 s^{-1}$ - fast. *(I)* The dynamics of V-PspF in the absence of σ^{54} is PspA-independent. The over-expression of plasmid borne PspA in Δ*rpoN* cells slightly changed the dynamics of V-PspF as shown by normalised distribution of diffusion coefficient of V-PspF in the absence (green, n=271) or in the presence of PspA (white, n=272). Inset: as in *(H)*.

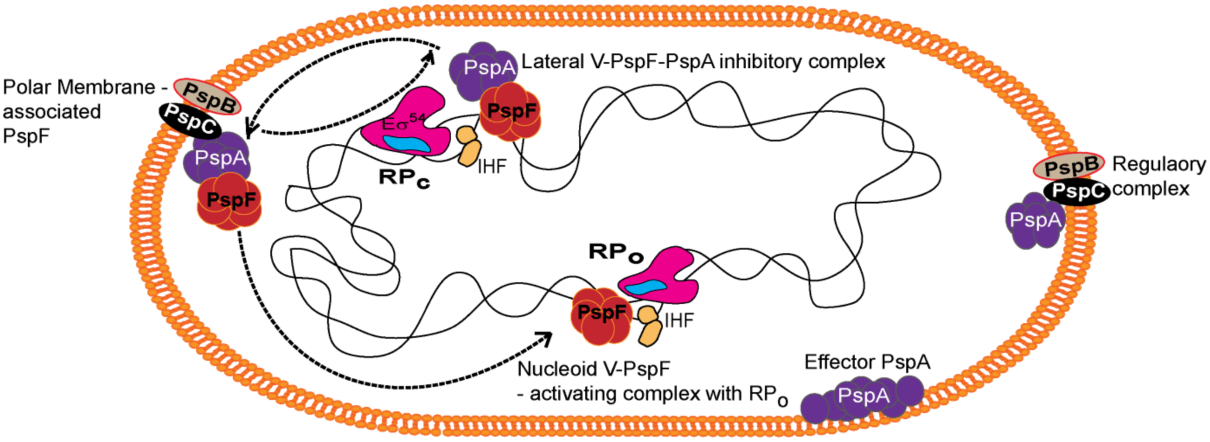

Supplementary Figure S7. Working model for key temporal, spatial and stoichiometric characteristics of the bacterial enhancer-dependent membrane stress response Phage shock protein (Psp) system of *E. coli*. The SMI study established the pivotal role of intracellular communication between the nucleoid-bound inhibitory complex of PspF-PspA and the membrane (IM PspBC sensors) with the on-set of stress. In the Psp system the bEBP PspF forms DNA and IM interacting complexes, the latter communicating information about membrane damage leading to the release of PspA negative control upon PspF, induction of *psp* genes, including the effector proteins PspA and PspG for final adaptation to the IM stress (see main text for details). Under stress, PspA switches from a regulatory hexamer to high order oligomer IM effector complexes but the mechanism, determinants and spatial and temporal organisation of these events at the IM are not well understood. Abbreviations: PspF, hexamer, bacterial enhancer binding protein (bEBP); PspA, hexamer, negative regulator of PspF; PspBC, inner membrane (IM, ocra) Psp sensors; PspA effector, high order oligomer (36mer), membrane stress controller; $E\sigma^{54}$, σ^{54}-RNA polymerase holoenzyme; IHF, integration host factor that bends DNA and brings in close proximity PspF and σ^{54}; RPc, closed promoter complex; RPo, open promoter complex.

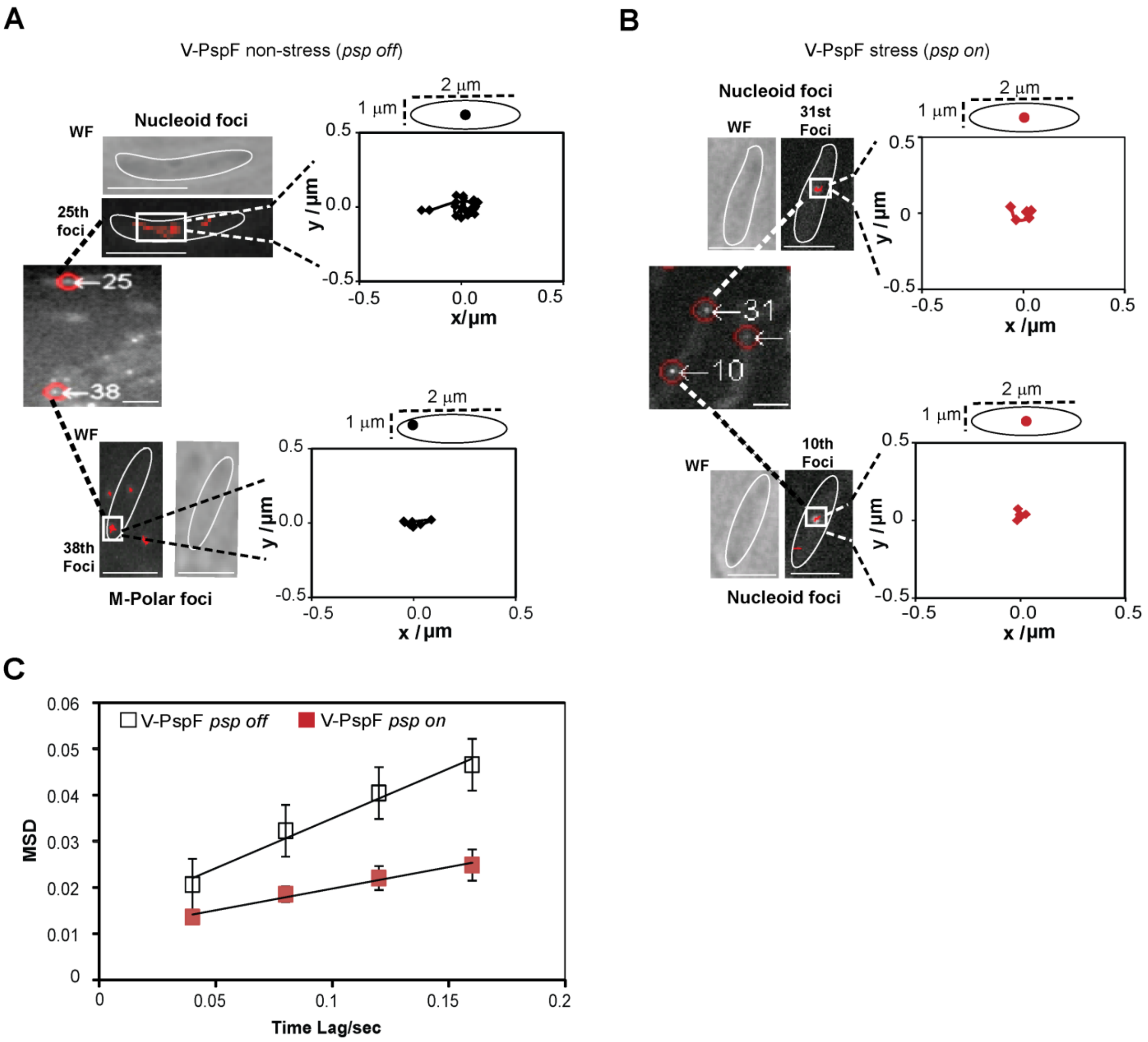

Supplementary Figure S8. Measuring the apparent two-dimensional diffusion coefficients (example for V-PspF *psp off*/*on* conditions). The schematic shows individual tracks obtained by the single molecule tracking algorithm and diffusion analysis[38] for V-PspF foci in cells under (*A*) non-stress (*psp off*) and (*B*) stress (*psp on*) conditions. The tracks for specific foci localised in central and polar (*psp off*) or central foci (*psp on*) were plotted with respect to *E. coli* cell dimensions (in µm). These tracks were used to generate MSD curve plots and diffusion coefficients for the foci. The images *A* and *B*, scale bar=1µM. (*C*) An example of a typical MSD curve plot (mean values with SD) obtained for V-PspF foci in non-stressed cells (white, n=82) compared to stressed cells (red, n=71) using this algorithm. The data are the mean values with SD error bars.

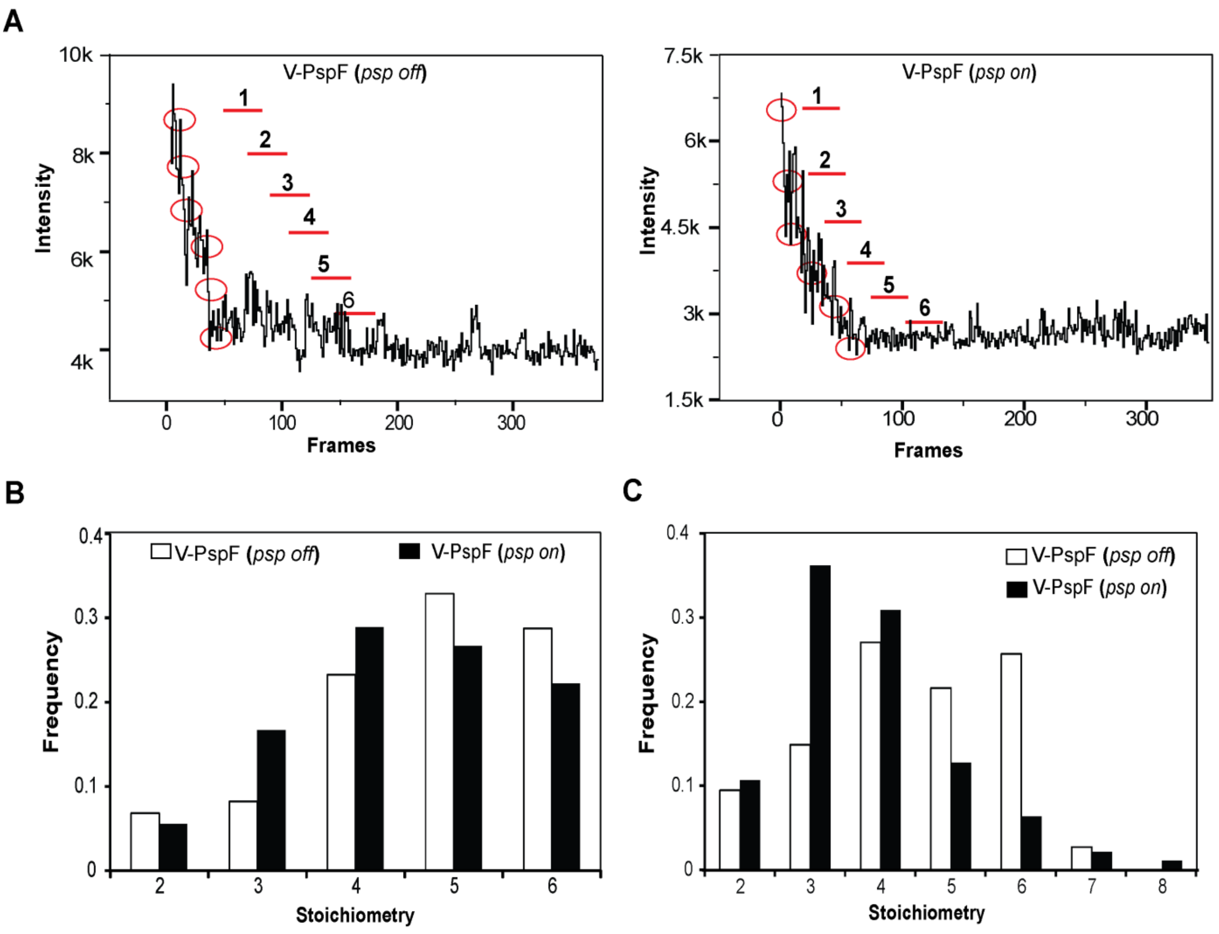

Supplementary Figure S9. Manual stoichiometry estimation. *(A)* Curves represent the bleaching steps of V-PspF under non-stress (*psp off*) and stress (*psp on*) growth conditions. Their manual analysis consolidates that of the edge preserving approach (see Fig. 4). Inspection of the bleaching steps reveals a single step corresponding to a monomer of V-PspF. *(B)* The distribution of stoichiometries calculated by manual counting of the bleaching and/or blinking steps for V-PspF foci [white, under non-stress (n=73) and black, under stress conditions (n=90)]. (*C*) The distribution of stoichiometries determined for each bleaching trace by dividing the estimated average step size with difference between the initial intensity and final intensity [white, under non-stress (n=75) and black, under stress conditions (n=94)].

Supplementary Table S1. *E. coli* K-12 strains and plasmids used in this study

Strain or plasmid	Relevant characteristics	Reference
**Strain**		
MG1655	WT	CGSC# 7740
MG1655 *pspF*::*nptI-sacB kan*	MG1655 chromosomal *pspF*::*nptI-sacB kan* (kan^r, $sucrose^s$)	This work<br>MG1655/ pTKRED(λRed) + *nptI-sacB kan*
MG1655 V-PspF	MG1655 expressing chromosomal Venus-PspF (kan^s, $sucrose^r$)	This work<br>MG1655 *pspF*::*nptI-sacB kan* /pTKRED(λRed) + *venus-pspF*
MG1655 V-$PspF^{W56A}$	MG1655Δ*pspF* expressing chromosomal Venus-$PspF^{W56A}$ mutant (kan^s, $sucrose^r$)	This work<br>MG1655 *pspF*::*nptI-sacB kan* /pTKRED(λRed) + *venus-pspF*^{W56A}
MG1655 V-PspF tet	MG1655 V-PspF *venus-pspF*::*tet* (tet^r)	This work<br>MG1655 V-PspF/pTKRED(λRed) + *venus-pspF*::*tet* (pTKS Tet template)
MG1655 V-PspFΔHTH	MG1655 expressing chromosomal Venus-$PspF_{1\text{-}275}$ (tet^s)	This work<br>MG1655 V-PspF tet/pTKRED(λRed) + *venus-pspF*$_{1\text{-}275}$(Stop276)
JWK3169	BW25113 Δ*rpoN*::Kan (kan^r)	39
MG1655 V-PspF Δ*rpoN*	MG1655 V-PspF Δ*rpoN*::Kan (kan^r)	This work<br>MG1655 V-PspF x P1/JWK3169
MC1068	MC1061 Δ*himA::Tn10* (tet^r)	Laboratory collection
MG1655 V-PspF Δ*himA*	MG1655 V-PspF Δ*himA*::*Tn*10 (tet^r)	This work<br>MG1655 V-PspF x P1/MC1068
MG1655 V-$PspF^{W56A}$ Δ*himA*	MG1655 V-$PspF^{W56A}$ Δ*himA*::*Tn*10 (tet^r)	This work<br>MG1655 V-$PspF^{W56A}$ x P1/MC1068
MVA129	MG1655 V-PspF ΔP_{pspG}::*kan* (kan^r)	This work<br>MG1655 V-PspF/pKD46(λRed1) + ΔP_{pspG}::*kan*(pKD4 Kan template)
MVA44	MG1655 ϕ(P*pspA-lacZ*) (amp^r)	40
MVA131	MVA44 ΔP_{pspG}::*kan* (amp^r kan^r)	This work<br>MG1655 V-PspF x P1/MVA129
C600	F^- tonA21 thi-1 thr-1 leuB6 lacY1 glnV44 rfbC1 fhuA1 λ^-	Laboratory collection
SA1943	*galK*$_{am}$ *his rpsL*	41
SA1943 λV-PspA	SA1943 *attB*::P_{pspA}-*venus-pspA* (amp^r)	This work<br>SA1943 x λBDC531(P_{pspA}-*venus-pspA* amp^r)
DY226	W3110 _*lac*U169 *gal*490 λ[*N*::*lacZ* *imm*λ(*cro-bio*)<br>*rnc*14λ^r	41
DY226 λV-PspA	DY226 *attB*::P_{pspA}-*venus-pspA* (amp^r)	This work<br>DY226 x P1/SA1943 λV-PspA
MG1655Δ*pspA*	MG1655 Δ*pspA*	15
MVA127	MG1655Δ*pspA* expressing chromosomal Venus-PspA (amp^r)	This work<br>MG1655Δ*pspA* × P1/DY226 λV-PspA
MG1655 *lacI::gfp*	MG1655 expressing chromosomal LacI-GFP fusion (kan^r)	A gift from S. Busby and M.A. Sánchez-Romero; 42
XL1-Blue	tet^r	Laboratory collection
**Plasmid**		
pTKRED	Plac λ-Red P*ara* I-SceI RecA (spc^r)	43
pTKS	*tetA*-I-*Sce*I recognition sites (tet^r)	43
pUM24	*nptI-sacB kan* cassette ($sucrose^s$, kan^r, amp^r)	A gift from T. Friedrich; 44

	cartridge plasmid	
pKD46	Red1 recombinase expressing plasmid (amp^{r})	45
pKD4	FRT-Kan-FRT cassette template plasmid (kan^{r})	45
pCS2	Plasmid expressing fast maturing fluorescent protein Venus (amp^{r})	A gift from T. Nagai
pCG65	Venus/pCS2 carrying Venus-PspA fusion (amp^{r})	This work
pRS415	Transcription fusion vector (amp^{r})	46
pCG66	pRS415 carrying P_{pspA}-Venus-PspA fusion (amp^{r})	This work
pCP20	*FLP*$^{+}$, λ *c*I857^{+}, λ p_R Rep^{ts}, (amp^{r}, cam^{r})	47
pGJ4	P_{lacUV5}-*gIV* (pIV) (tet^{r})	48
pPB8-WT	pBAD18-cam expressing PspF WT (cam^{r})	49
pPB8-W56A	pBAD18-cam expressing PspF^{W56A} (cam^{r})	49
pPB10	pBAD18-cam expressing PspA WT (cam^{r})	40
pAJM3	pBAD18-cam expressing PspBC (cam^{r})	23
pPB9	pAPT110 expressing PspA WT (kan^{r} spc^{r})	49
pSJ1	pMR25 carrying ϕ(P*pspA-lacZ*) (tet^{r})	50
pLL1	pMC1403 carrying ϕ(P*pspG-lacZ*) (amp^{r})	15
pCE1	pDSW210 based σ^{54}-YFP fusion (amp^{r})	This work *rpoN* gene fused to mutagenized eGFP to make YFP
pGEM-T Easy	Cloning vector (amp^{r})	Promega

Supplementary Methods

Construction of strains expressing chromosomal V-PspF and its derivatives. The strains expressing Venus-(linker)-PspF (V-PspF) and Venus-(linker)-PspF^{W56A} (V-PspF^{W56A}) were constructed using the Red recombineering method for gene replacement[43,45] and antibiotic/sucrose counter-selection using the (*nptI-sacB)* system[51]. The *pspF* gene in MG1655 was first replaced with the *nptI-sacB kan* cassette, with kanamycin resistant/sucrose sensitivity selection and later replaced with *venus*-linker-*pspF*/*pspF*^{W56A} fusions selecting for resistance to 5% sucrose and kanamycin sensitivity (see Supplementary Table S1). The overlap PCR method was used to place together *venus* and *pspF*/*pspF*^{W56A} genes with a linker (-gaa ttc acc aga acc acc gaa ttc acc aga acc acc). For the overlap PCR reaction the Pfu ultra II (HS) (Stratagene) polymerase, the *pspF or pspF*^{W56A} 3' flanking and *pspF* 5' region primers with the pPB8-WT or pPB8-W56A template respectively, and the 5' *pspF* region/3' *venus* and *venus* 5' hybrid primers with the pCS2 template were used to produce *venus-(linker)-pspF*/*pspF*^{W56A} DNA fragments.

In the first step, the allelic exchange was performed between the MG1655 chromosomal *pspF* and the PCR amplified [Pfu ultra II (HS) (Stratagene)] *nptI-sacB kan* cassette containing 5' and 3' *pspF* flanking DNA sequences by using kanamycin(resistance)-sucrose (sensitivity) counter-selection. The MG1655 carrying pTKRED (helper plasmid expressing the Red recombinase; spectinomycin resistant, 100 µg ml^{-1}) was transformed with the *nptI-sacB kan* linear DNA cassette by electroporation. The electroporation was carried out with a

1 mm gap cuvettes and Ec1 setup on Bio-Rad micropulser; after electroporation cells were recovered for 3 hr in SOC media at 30°C. The transformants were selected on LA+kanamycin (25 μg ml^{-1}) plates at 42°C to eliminate pTKRED plasmid (temperature sensitive). The kanamycin-resistant colonies, arising by integration of the *nptI-sacB kan* cassette into the *pspF* gene on the chromosome of strain MG1655, were grown overnight in liquid culture without selection and plated onto glucose and then by replica plating onto sucrose plates to select for sucrose-sensitive candidates. The colonies were checked by performing PCR using flanking primers specific for the *nptI-sacB kan* cassette.

In the second step, MG1655 *pspF*::*nptI-sacB kan* now carrying pTKRED where transformed by *venus-(linker)-pspF* or *venus-(linker)-pspF*^{W56A} linear DNA by electroporation and the *nptI-sacB kan* has been excised from the chromosome through a second recombination event being replaced by *venus-(linker)-pspF/pspF*^{W56A}. The desired sucrose-resistant kanamycin sensitive phenotype of candidate recombinants (MG1655 V-PspF or MG1655 V-PspF^{W56A}; see Supplementary Table S1) was verified by replica-plating and candidate colonies were screened for the desired genotype by colony PCR with primers designed to anneal outside the sites of recombination. PCR products were sequenced to check that the Venus-(linker)-PspF or Venus-(linker)-PspF^{W56A} was correctly inserted and for point mutations.

To obtain MG1655 V-PspFΔHTH expressing Venus-(linker)-PspF$_{1-275}$, the same recombineering method and the pTKRED were used to replace the V-PspF with the V-PspF$_{1-275}$, except that tetracycline resistance cassette containing the *pspF* flanking primers (PCR amplified from the plasmid pTKS) was first introduced into the MG1655 V-PspF recipient (MG1655 V-PspF tet) and then in the second step replaced with the Venus-(linker)-PspF$_{1-275}$ and selected for the tetracycline sensitive MG1655 V-PspFΔHTH recombinants (see Supplementary Table S1). The V-PspF from MG1655 V-PspF, the PCR based mutagenesis and the subsequent PCR amplification was used to generate a linear DNA with stop codon introduced at the position coding for the amino acid 276th in PspF, to express V-PspF$_{1-275}$.

Construction of strain expressing chromosomal V-PspA. The Venus-(linker)-PspA (Venus-PspA; V-PspA) was expressed as an N-terminal fusion under control of the native *pspA* promoter integrated as a single copy into the *attB* site of the *E. coli* chromosome of Δ*pspA* strain (Supplementary Fig. S1D). The V-PspA fusion was constructed by cloning the (linker: ggt ggt ggt ggt tct ggt ggt tct gag ttc)-*pspA* DNA *Eco*RI-*Xba*I fragment into Venus expressing plasmid pCS2 and then excising the Venus stop codon and *Eco*Ri site using site specific mutagenesis, creating pCG65 (Supplementary Table S1). The P$_{pspA}$-*venus-pspA* (Venus-PspA fusion under control of the *pspA* promoter) was constructed by separately amplifying 0.3 kb of the *pspA* promoter region as an *EcoRI-NcoI* fragment from MG1655 *E.*

coli genomic DNA and 1.3 kb of the *venus*-(*linker*)-*pspA* fusion *Nco*I-*Bam*HI fragment from pCG65, ligating, and then subcloning 1.6 kb *Eco*RI-*Bam*HI fragment into pRS415[46], creating pCG66 (Supplementary Table S1). This construct was verified by sequencing. The single-copy chromosomal fusion of P_{pspA}-*venus*-*pspA* was constructed as described[41] (see Supplementary Table S1). Briefly, phage λBDC531 (*imm*[21]) was grown in a C600 strain carrying pCG66. The resulting λ phages were used to generate lysogens in SA1943 and screened for a Gal^- phenotype on MacConkey galactose agar plates supplemented with ampicillin (40 μg ml^{-1}). P1 phage was grown on the SA1943 λV-PspA lysogens and the resultant lysates used to transduce the recipient strain DY226, which was screened for ampicillin resistant transductants at 42°C. The constructed strain carrying the P_{pspA}-*venus*-*pspA* fusion inserted into *attB* was then screened for the Bio^+ phenotype and the presence of prophage (sensitivity to λ phages). Finally, P_{pspA}-*venus*-*pspA* fusion was moved by $P1_{vir}$ transduction[36] into strain MG1655Δ*pspA*, generating ampicillin resistant strain MVA127.

Construction of a strain lacking the *pspG* regulatory region. A deletion of the *pspG* promoter (ΔP_{pspG}) in MG1655 V-PspF cells was constructed using Nano Bio protocol for gene knockout based on method developed by Datsenko and Wanner[45] (see Supplementary Table S1). We used hybrid primers containing the flanking sequences of the *pspG* promoter region and sequences homologous to 5' or 3' of the FRT-Kan-FRT cassette placed on the template plasmid pKD4 to amplify (Promega Pfu polymerase and the corresponding buffers) 1.1 kb linear DNA fragment from pKD4 that substitutes for the 226 bps long P_{pspG} on the chromosome. Target strain MG1655 V-PspF was transformed by pKD46 expressing the Red recombinase and cells were grown at 30°C. The amp^r transformants were selected and electroporated using the linear DNA PCR product. The electroporation was carried out with 1 mm gap cuvettes and Ec1 setup on Bio-Rad micropulser. After electroporation cells were recovered for 3 hr in SOC media at 37°C and grown on LB+kan (25 μg ml^{-1}) plates for 24 hr at 42°C [loss of temperature sensitive pKD46 (Red1)] and then the kan^r and subsequently kan^r amp^s recombinants were selected. The presence of FRT-Kan-FRT replacing the P_{pspG} was verified with colony PCR using pair of primers containing the flanking sequences of the *pspG* promoter region and the PhusionTaq polymerase (Fermentas) and the verified strain was named MVA129. The antibiotic cassette was not removed with pCP20 allowing $P1_{vir}$ transduction[1] to move ΔP_{pspG}::Kan into strains of interest.

Microscopy and data analysis. The diffusion analysis was performed as previously described[37] using Matlab (Mathwork) scripts based on the algorithm by Crocker and Grier[38]. The analysis of dynamics of imaged foci is based on localising the particles (between 200-1000 foci tracks) with sub-pixel accuracy, joining the localisations to tracks and calculating

the mean square displacement (MSD) for different time lags (Supplementary Fig. S8). The diffusion coefficient for each track is calculated as 1/4th of the slope of the MSD curve.

We followed the method described previously[25] to measure the stoichiometry of protein complexes in living cells. In brief, an edge-preserving non-linear digital filter based on Chung-Kennedy algorithm (see below) was first applied to the photobleaching intensity trace in order to effectively discard additive Gaussian-like noise. The output of this filter is a smoother and clearer signal, with an improved SNR. This signal was subsequently used to calculate the PDDF (Pairwise Difference Distribution Function; see below) and its power spectrum. Finally, peaks in the power spectrum are detected and the number of steps present in the original photobleaching trace is determined.

Chung-Kennedy Algorithm

The Chung-Kennedy Algorithm[48,52] is a noise-removal filter that preserves the steps in step-wise signals. A forward and backward window is generated from any given data point to sample the same number of points before and after the point from which the windows are generated. The number of points sampled (i.e. the window size) is variable. The mean and variance of each window is calculated and the output (filtered) value is the one with the smaller variance. This preserves steps as the variance of a window straddling points on both sides of a step-change will be larger than a window with points on only one side. The window size is selected to give the smallest standard deviation between the raw and filtered signals. Only one pass of the filter was applied to the data.

Pairwise Difference Distribution Function

The pairwise difference distribution function (PDDF)[53,54] for each filtered bleaching trace was calculated as follows. For every data point, its value is subtracted from all data points that follow. For a signal having n data points, the total number of pairwise differences will be equal to $n(n - 1)/2$. Subsequently the distribution of these pairwise differences can be calculated using a user-defined number of bins, in this case 2000. The PDDF is then normalised by dividing each bin count with the total number of pairwise differences to give the percentage of the total number of pairwise differences in each bin. The power spectrum of the PDDF was then calculated by fast Fourier transform using the Hanning window.

Photobleaching of individual V-PspF foci from non-stressed and stressed cells was also determined using manual counting methods (Supplementary Fig. S9A). The numbers of bleaching steps were counted manually (blinking of the Venus fluorescence was taken into account) (Supplementary Fig. S9B) and the distribution data were confirmed by calculating the average step size from the data to estimate stoichiometry (Supplementary Fig. S9C). The data was collected for foci using edge preserving algorithm (see Fig. 4) and manual counting of bleaching steps methods, and distribution of the stoichiometries was plotted. The stoichiometry estimation of self assemblies *in vivo* is complicated and requires careful

attention[9] so rigorous data analysis is crucial for stoichiometric estimations. Therefore we used the three methods (above and in the main text) to independently analyse the photobleaching data.

Supplementary References

38. Crocker, J. C. & Grier, D. G. Methods of digital video microscopy for colloidal studies. *J. of Colloid and Interface Sci.* **179**, 298-310 (1996).

39. Baba, T., Ara, T., Hasegawa, M., Takai, Y., Okumura, Y., Baba, M., Datsenko, K. A., Tomita, M., Wanner, B. L. & Mori, H. Construction of *Escherichia coli* K-12 in-frame, single-gene knockout mutants: the Keio collection. *Molecul. Systems Biol.* **2**, 2006.0008 (2006).

40. Jovanovic, G., Lloyd, L. J., Stumpf, M. P. H., Mayhew, A. J. & Buck, M. Induction and function of the phage shock protein extracytoplasmic stress response in *Escherichia coli*. *J. Biol. Chem.* **281**, 21147–21161 (2006).

41. Yu, D. & Court, D. L. A new system to place single copies of genes, sites and *lacZ* fusions on the *Escherichia coli* chromosome. *Gene* **223**, 77-81 (1998).

42. Sánchez-Romero, M-A., Lee, D. J., Sánchez-Morán, E. & Busby, J. W. Location and dynamics of an active promoter in *Escherichia coli* K-12. *Biochem. J.* **441**, 481-485 (2012).

43. Kuhlman, T. E. & Cox, E. C. Site-specific chromosomal integration of large synthetic constructs. *Nuc. Acids Res.* **38**, e92 (2010).

44. Pohl, T., Uhlmann, M., Kaufenstein, M. & Friedrich, T. Lambda Red-mediated mutagenesis and efficient large scale affinity purification of the *Escherichia coli* NADH:ubiquinone oxidoreductase (complex I). *Biochemistry* **46**, 10694-10702 (2007).

45. Datsenko, K. A. & Wanner, B. L. One-step inactivation of chromosomal genes in *Escherichia coli* K-12 using PCR products. *Proc. Natl. Acad. Sci. U.S.A.* **97**, 6640-6645 (2000).

46. Simons, R. W., Houman, F. & Kleckner, N. Improved single and multicopy *lac*-based cloning vectors for protein and operon fusions. *Gene* **53**, 85-96 (1987).

47. Cherepanov, P. P. & Wackernagel, W. Gene disruption in *Escherichia coli*: Tc^R and Km^R cassettes with the option of Flp-catalyzed excision of the antibiotic-resistance determinant. *Gene* **158**, 9-14 (1995).

48. Leake, M. C., Chandler, J. H., Wadhams, G. H., Bai, F., Berry, R. M. & Armitage, J. A. Stoichiometry and turnover in single, functioning membrane protein complexes. *Nature* **443**, 355-358 (2006).

49. Elderkin, S., Bordes, P., Jones, S., Rappas, M. & Buck, M. Molecular determinants for PspA-mediated repression of the AAA transcriptional activator PspF. *J. Bacteriol.* **187**, 3238–3248 (2005).

50. Jones, S. E., Lloyd L. J., Tan, K. K. & Buck, M. Secretion defects that activate the phage shock response of *Escherichia coli*. *J. Bacteriol.* **185**, 6707-6711 (2003).

51. Ried, J. L. & Collmer, A. An *npt*I-*sacB*-*sacR* cartridge for constructing directed, unmarked mutations in gram-negative bacteria by marker exchange-eviction mutagenesis. *Gene* **57**, 239-246 (1987).

52. Chung, S. H. & Kennedy, R. A. Forward-backward non-linear filtering technique for extracting small biological signals from noise. *J. Neurosci. Methods* **40**, 71-86 (1991).

53. Kuo, S. C., Gelles, J., Steuer, E. & Sheetz, M. P. A model for kinesin movement from nanometer level movements of kinesin and cytoplasmic dynein and force measurements. *J. Cell Sci.* **14**, 135-138 (1991).

54. Lenn, T., Leake, M. C. & Mullineaux, C. W. Clustering and dynamics of cytochrome bd-I complexes in the *Escherichia coli* plasma membrane *in vivo*. *Mol. Micobiol.* **70**, 1397-1407 (2008).
